# Structures and ammonia synthesis activity of hexagonal ruthenium iron nitride phases

**DOI:** 10.1016/j.isci.2024.110795

**Published:** 2024-08-23

**Authors:** Li Shao, Angela Daisley, Michael Higham, C. Richard A. Catlow, Justin S.J. Hargreaves, Andrew L. Hector

**Affiliations:** 1 School of Chemistry, University of Southampton, Southampton SO17 1BJ, UK; 2 School of Chemistry, Joseph Black Building, University of Glasgow, Glasgow G12 8QQ, UK; 3 Department of Chemistry, University College London, 20 Gordon Street, London, UK; 4 Research Complex at Harwell, Rutherford Appleton Laboratory, Harwell Oxford, Didcot, Oxon OX11 0FA, UK; 5 School of Chemistry, Cardiff University, Park Place, Cardiff, UK

**Keywords:** Chemistry, Chemical reaction, Materials science

## Abstract

A series of ruthenium iron nitride phases with Ru:Fe ratios of ca. 1:3 were synthesized by ammonolysis. When the ammonolysis temperature was above 500°C, the obtained Ru_x_Fe_3_N_y_ materials had a ε-Fe_3_N (*P*6_3_22) structure, while two similar phases were present when the ammonolysis was lower than 500°C. Powder neutron diffraction identified one phase as relating to the ε-Fe_3_N structure, while the other had a disordered NiAs-type (*P*6_3_/*mmc*) structure. These ternary metal nitrides show ammonia synthesis activity at low temperature (200°C–300°C) and ambient pressure, which can be related to the loss of lattice nitrogen. Steady state catalytic performance at 400°C is associated with ruthenium-iron alloy. Additionally, density functional theory calculations were performed using an approximate model for the disordered hexagonal phase, revealing that this phase is more stable than a cubic anti-perovskite phase which has been previously investigated computationally, and corroborating the experimental findings of the present work.

## Highlights

• Samples consist of ε-Fe_3_N *P*6_3_22 and disordered hcp (*P*6_3_/*mmc*) structures
• Fe_3_RuN-500°C has high selectivity for conversion of lattice nitrogen to NH_3_
• Steady state activity at 400°C is associated with Fe-Ru alloy
• The chemical looping capabilities of the nitride appears to be limited

## Graphical abstract

**Figure:**
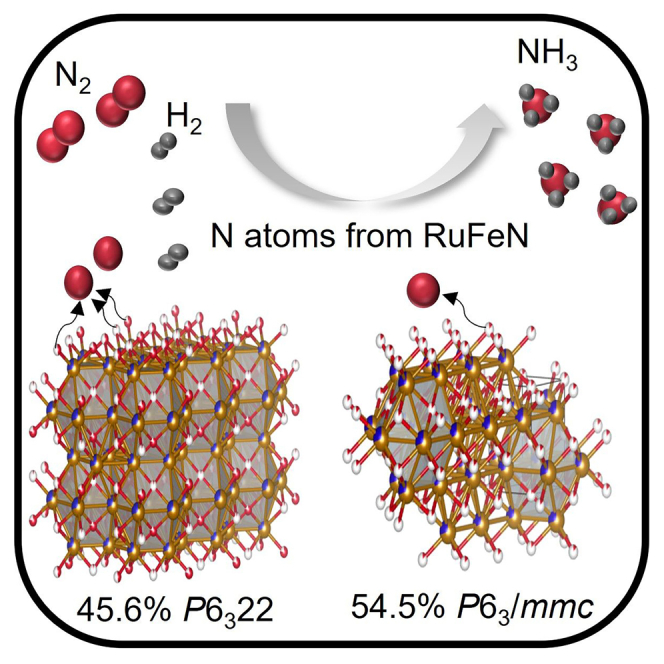

## Introduction

Ammonia which is produced on the industrial scale by the Haber Bosch process, is an important feedstock for the production of fertilizer. Accordingly, the production of ammonia via the Haber-Bosch process is credited with the sustenance of 40% of the global population.^1^ This process combines H_2_ and N_2_ into ammonia with fused-iron catalysts at high temperature (400°C–600°C) and high pressure (20–40 MPa).^2^ However, the process consumes 1–2% of the world’s energy supply^3^ and produces significant carbon emissions.^4^ Therefore, developing active catalysts that produce ammonia effectively under lower pressure and temperature conditions is one of the most important and challenging topics for the Haber-Bosch process. Limitations to the development of highly active metal catalysts relate to so called scaling relationships in which there is an optimum nitrogen binding energy associated with optimal performance.^5^ This idea has led to the development of the active Co_3_Mo_3_N catalyst in which the combination of Co (which activates N_2_ weakly) with Mo (which activates N_2_ strongly) as expressed in the surface (111) plane and ordered by the presence of lattice N leads to high activity.^6^^,^^7^^,^^8^^,^^9^ An alternative explanation for the performance of this catalyst relates to the occurrence of an N based Mars-van Krevelen mechanism^10^ associated with the presence of surface N vacancies and possibly acting via an associative mechanism in which hydrogenation of activated N_2_ occurs prior to N-N bond dissociation.^11^^,^^12^^,^^13^ Ammonia synthesis can also be accomplished from Co_3_Mo_3_N via a chemical looping mechanism wherein reduction to Co_6_Mo_6_N liberates some NH_3_ with Co_3_Mo_3_N being regenerated by N_2_ alone.^10^ Accordingly, it is of interest to investigate metal nitrides as both catalysts and also looping reagents in the context of ammonia production.

In our previous work, binary, ternary, and quaternary metal nitrides have been fabricated and investigated for ammonia synthesis activity under ambient pressure. For example, Ni_2_Mo_3_N and NiCoMo_3_N were produced using a modified Pechini route and Ni_2_Mo_3_N showed good activity for ambient pressure ammonia synthesis.^14^^,^^15^ (Ni,M)_2_Mo_3_N (M = Cu or Fe) were produced using a citrate gel route and showed ammonia synthesis activity at 500°C and ambient pressure.^16^

Studies have been extended toward anti-perovskite nitrides for which it might be possible to tune performance by controlled composition. However, to date the anti-perovskite nitrides investigated—Co_3_ZnN,^17^ Ni_3_ZnN,^17^ Co_3_InN,^17^ Ni_3_InN,^17^ Co_3_CuN^18^ and Ni_3_CuN^18^—were observed to produce ammonia only due to the loss of lattice nitrogen. In view of the established catalytic performance of Fe and Ru for ammonia synthesis, in this context the performance of RuFe_3_N is of interest.^4^^,^^19^ Theoretical investigations have discussed the structural, elastic, magnetic, and electronic properties of cubic anti-perovskite RuFe_3_N,^20^^,^^21^^,^^22^^,^^23^^,^^24^ suggesting that RuFe_3_N in γ′-Fe_4_N structure exhibits metallic behavior, with a finite density of electronic states at the Fermi level.^24^ However, anti-perovskite RuFe_3_N has not so far been synthesized, and its structural and catalytic properties have not been investigated. In this study, hexagonal ternary metal nitrides with compositions close to RuFe_3_N are fabricated via a citrate-gel route with different compositions and their activity for ammonia synthesis is investigated at ambient pressure and at temperatures between 200°C and 400°C.

## Results

### Characterization of the ruthenium iron nitrides

A series of ruthenium iron nitrides with compositions close to RuFe_3_N were prepared by a citrate-gel method. The results show that the Ru:Fe molar ratio in the precursor solution, ammonolysis temperature, and ammonolysis duration, have an impact on the composition of the products. Table 1 reports the synthetic procedure undertaken for the samples, and also presents the nitrogen content in the final products and sample compositions expressed as Ru_x_Fe_3_N_y_. Nitrogen mass fractions were obtained from combustion analysis. The Ru and Fe molar ratio in the solution and in the final products were obtained from energy-dispersive X-ray spectroscopy (EDX). It can be seen that the Ru content in the final product is apparently slightly less than that expected on the basis of the synthesis ratio which possibly relates to evaporation of some Ru during ammonolysis. For ratios of around Ru:Fe = 1.4:3 in solution, the Ru:Fe in the final product is close to that of the target ratio for the anti-perovskite phase (1:3). Two additional materials were also prepared as outlined in Tables S1 and S4 and Figure S2.

**Table 1:** Synthetic process of ruthenium iron nitrides, nitrogen mass fractions in samples and sample compositions expressed as Ru_x_Fe_3_N_y_

Sample No.	Ru: Fe molar ratio in solution	Ammonolysis		Nitrogen content (wt. %)	Composition expressed as Ru_x_Fe_3_N_y_
		Temperature/°C	Duration/h		
1	0.6:3	900°C	12 h	6.42%	Ru_0.51_Fe_3_N_1.07_
2	1.0:3	900°C	12 h	4.22%	Ru_0.86_Fe_3_N_0.80_
3	1.4:3	900°C	12 h	2.90%	Ru_0.93_Fe_3_N_0.56_
4	1.6:3	900°C	12 h	2.07%	Ru_1.11_Fe_3_N_0.42_
5	1.0:3	600°C	12 h	4.17%	Ru_0.82_Fe_3_N_0.78_
6	1.0:3	500°C	24 h	5.13%	Ru_0.77_Fe_3_N_0.97_
7	1.4:3	500°C	24 h	5.26%	Ru_1.03_Fe_3_N_1.08_
8	1.5:3	500°C	24 h	3.70%	Ru_1.20_Fe_3_N_0.79_
9	1.6:3	500°C	24 h	3.05%	Ru_1.96_Fe_3_N_0.82_
10	1.4:3	400°C	24 h	5.78%	Ru_1.12_Fe_3_N_1.23_
11	1.4:3	500°C	24 h	5.26%	Ru_1.03_Fe_3_N_1.08_
12	1.4:3	600°C	24 h	2.60%	Ru_1.07_Fe_3_N_0.53_
13	1.4:3	700°C	24 h	2.40%	Ru_1.13_Fe_3_N_0.49_
14	1.4:3	800°C	24 h	2.49%	Ru_1.04_Fe_3_N_0.50_
15	1.4:3	900°C	24 h	2.69%	Ru_0.96_Fe_3_N_0.52_

Samples are referred to Ru_x_Fe_3_N_y,_ followed by the ammonolysis temperature and duration.

Samples 7 and 11 are the same material. This is listed twice to emphasize “Ru and Fe molar ratio” control and “ammonolysis temperature control”, respectively.

**Figure 1:**
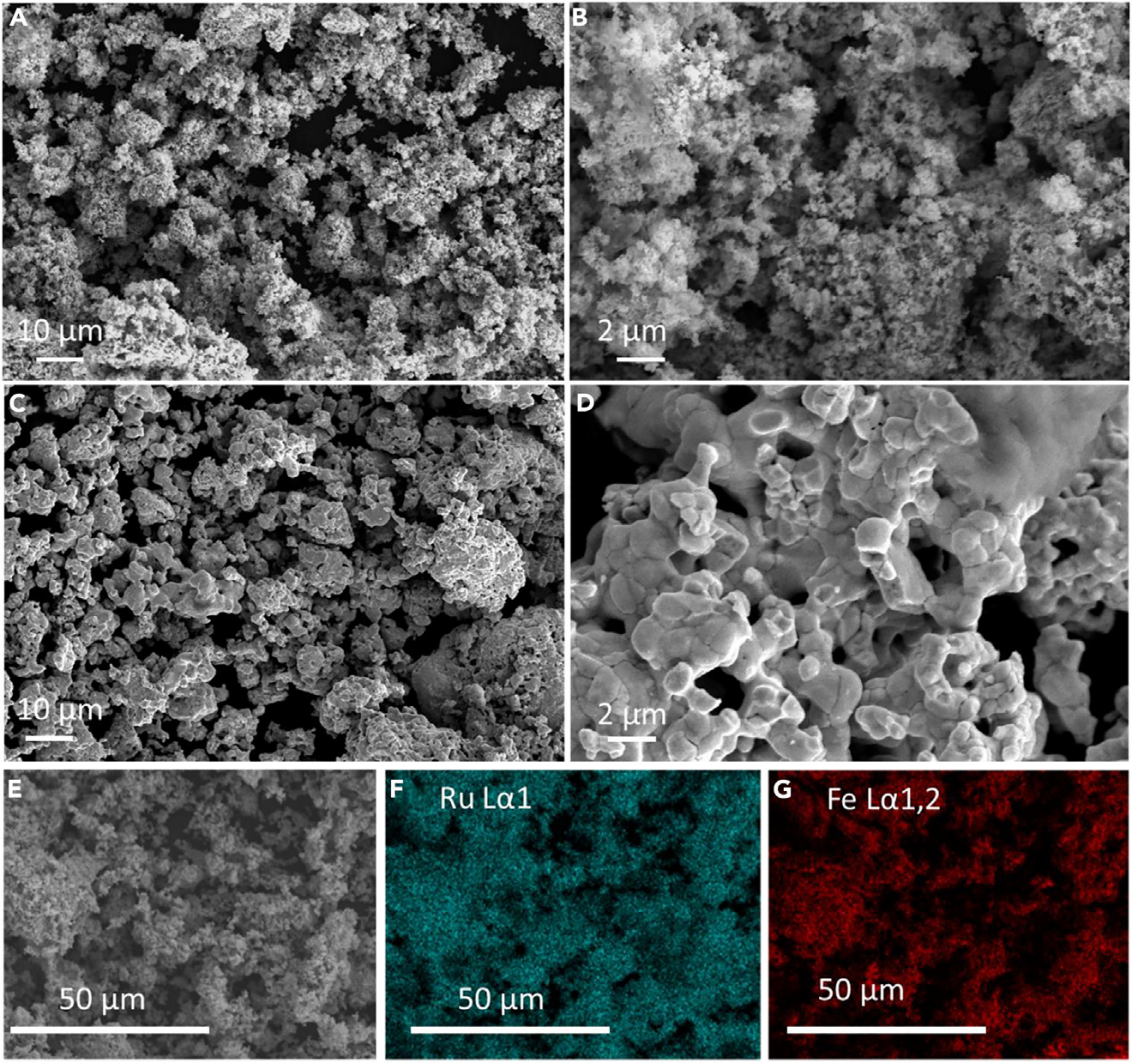
Characterisation of the ruthenium iron nitrides by SEM (A, B, and E) SEM images of Ru_1.03_Fe_3_N_1.08_-500°C-24 h. (C and D) SEM images of Ru_0.96_Fe_3_N_0.52_- 900°C-24 h. (F and G) EDX mapping images from Ru_1.03_Fe_3_N_1.08_-500°C-24 h SEM image in (E).

Figure 1 presents typical SEM images of sample Ru_1.03_Fe_3_N_1.08_-500°C-24 h (A,B) and Ru_0.96_Fe_3_N_0.52_- 900°C-24 h (C,D). Ru_1.03_Fe_3_N_1.08_ obtained at 500°C is comprised of small irregular particles, forming a rough surface, whereas Ru_0.96_Fe_3_N_0.52_ obtained at 900°C has interconnected and rounded particles presenting a smoother surface. Panels (F) and (G) illustrate the distribution of elements Ru and Fe, respectively, with in sample Ru_1.03_Fe_3_N_1.08_-500°C-24 h (shown in panel [E]), revealing that Ru and Fe are uniformly distributed throughout the particles.

**Figure 2:**
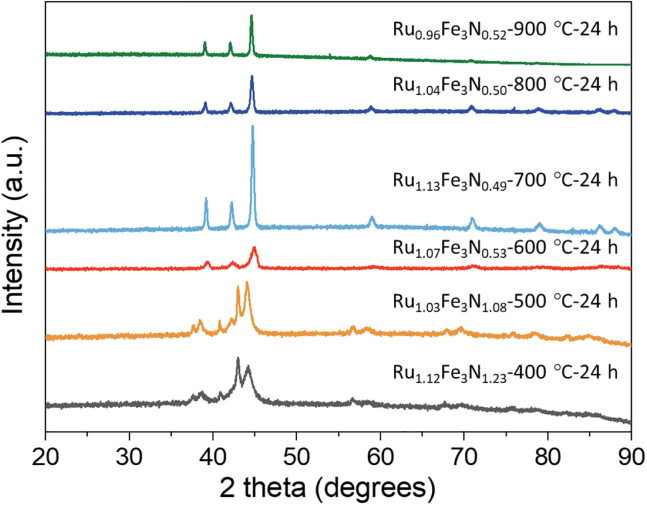
Characterisation of the ruthenium iron nitrides by XRD XRD patterns of the Ru_x_Fe_3_N_y_ samples prepared under different ammonolysis temperature ranging from 400°C to 900°C. The ruthenium and iron molar ratio in the precursor solution is 1.4:3. The ammonolysis duration was 24 h.

In Figure 2, the XRD patterns for Ru_x_Fe_3_N_y_ samples produced at ammonolysis temperatures ranging from 400°C to 900°C are presented. For ammonolysis temperatures below 500°C, the reflections are generally broad. The additional peaks observed between 40° and 45°, which are close to the intense peak at 44°, are possibly indicative of the presence of more than one phase. In contrast, samples produced by ammonolysis at 600°C and higher, correspond with the single-phase pattern for ε-Fe_3_N (space group *P*6_3_22). In all the samples prepared there is no evidence of the successful preparation of the anti-perovskite RuFe_3_N phase under the reaction conditions. Compared with the standard diffraction pattern of ε-Fe_3_N (ICSD-79981), the diffraction peaks of samples prepared within this study are shifted to higher 2θ values. The refined lattice parameters as well as the nitrogen content of the various samples prepared can be found in Table S2. Since the expected stoichiometric nitrogen value for RuFe_3_N is 4.94 wt. % it can be seen that samples prepared by ammonolysis at 500°C and below possess nitrogen contents in excess of this theoretical value, whereas for temperatures over 500°C, the nitrogen content drops significantly below it.

As can be seen from Figure S1 and Table S3, increasing the ruthenium content in the precursor solution decreases the nitrogen content in the final products significantly, as well as the lattice parameters. Two ε-Fe_3_N phases with different lattice parameters were used to refine the XRD patterns of the samples obtained at 500°C in Figure S1A, except for that of Ru_0.77_Fe_3_N_0.97_-500°C-24 h for which the second phase was at too low a concentration to fit. For the other three samples, the phase with smaller lattice parameters does not change significantly with an increase of ruthenium and their lattice parameters and phase ratios are similar. In the phase with the larger lattice parameters, a decreasing linear trend in lattice parameters with ruthenium content is observed.

From the previous analysis, it can be concluded that low ammonolysis temperature and low ruthenium content result in higher nitrogen content in the resulting ternary metal nitrides. When the ammonolysis temperature is 500°C or lower, two phases emerge, with one adopting *P*6_3_22 structure and for this phase larger lattice parameters correspond to a higher nitrogen content in the structure. In order to gain more understanding of the structural parameters in relation to the lattice nitrogen in samples, powder neutron diffraction (PND) studies have been undertaken.

### Powder neutron diffraction

**Figure 3:**
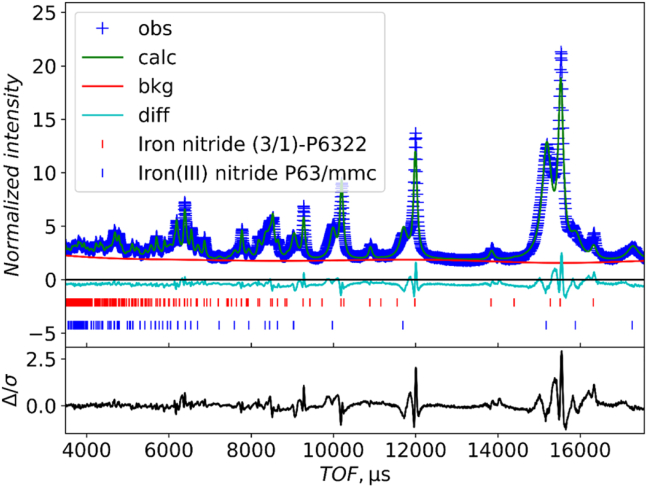
Powder neutron diffraction (PND) pattern of Ru_1.03_Fe_3_N_1.08_-500°C-24 h fitted with two phases, a ε-Fe_3_N type *P*6_3_22 structure (red) and a disordered hcp (*P*6_3_/*mmc*) structure (blue) Full details of refined parameters are in Table S6.

As noted previously, the XRD patterns of ruthenium iron nitride samples produced at 500°C show two hexagonal phases. Up to this point, both were modeled as the ε-Fe_3_N (space group *P*6_3_22) phase, but the phase with the smaller hexagonal lattice parameters had some reflections missing compared with this structure model. Ru_1.03_Fe_3_N_1.08_-500°C-24 h was characterized by PND to allow a more detailed structural study, and crucially to probe nitrogen occupation. The obtained PND pattern is displayed in Figure 3.

**Figure 4:**
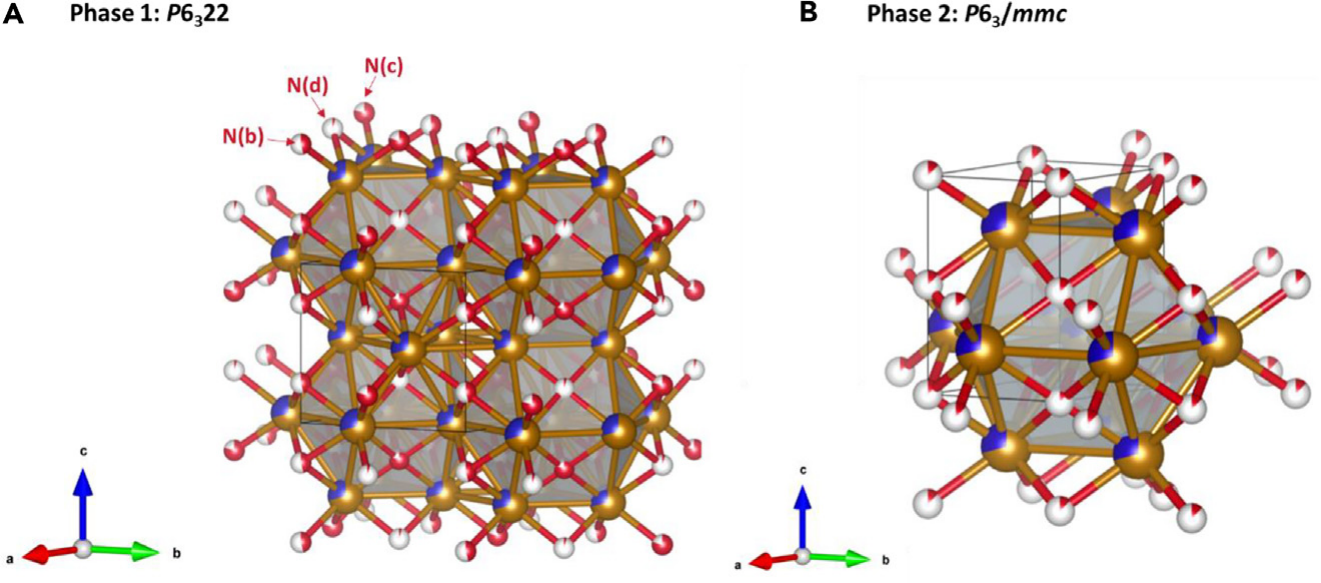
Perspective view of the unit cells of Ru_1.03_Fe_3_N_1.08_-500°C-24 h, constructed based on the results of Rietveld refinement (A) ε-Fe_3_N (*P*6_3_22) structure. (B) disordered hcp (*P*6_3_/*mmc*) structure. Blue: ruthenium, gold: iron and red: nitrogen. In phase *P*6_3_22 structure, the N(c) sites have high occupancy levels, N(b) sites have half occupancy and N(d) sites have very low occupancy.

Two ε-Fe_3_N type *P*6_3_22 phases with different lattice parameters were used as the starting model for the refinement and details are given in Figure S3 and Table S5. In both phases, the Ru and Fe metal atoms occupy the same position in the hexagonal close packed crystal matrix, with the smaller nitrogen atoms occupying the interstitial sites between the larger metal atoms in the crystal lattice.^25^ Compared to the ideal *P*6_3_22 Fe_3_N structure (ICSD-79982), some nitrogen atoms in these two phases distort from the N(c) site to the N(b) site, as shown in Figure 4A. This nitrogen distortion has also been reported for Ni_3_N.^25^ However, this two ε-Fe_3_N phase model does not fully account for the observed PND pattern since peaks at 1700 μs and 14500 μs are missing, and furthermore, the overall fitting of the peak intensities is poor. Therefore, a new model was considered.

The model of a ε-Fe_3_N (*P*6_3_22) phase and a phase with hexagonal close packed metal atoms and disordered nitrogen (model based on NiAs in *P*6_3_/*mmc* but with cation and anion positions reversed) provided a better fit to the phase with the smaller lattice parameters. Figure 3 shows the PND pattern of Ru_1.03_Fe_3_N_1.08_-500°C-24 h. The ε-Fe_3_N structure (red) and a disordered hcp structure (blue) were used to fit and refine the pattern, which clearly allowed a much-improved fit. The refined space group parameters and phase fractions are presented in Table 2. The full details of the refined parameters are given in Table S6. The perspective view of the unit cells is given in Figure 4. The set of high intensity and sharp peaks correspond to the ε-Fe_3_N type phase (*P*6_3_22), with a 45.6% phase fraction. The set of lower intensity and broader peaks belong to the disordered hcp (*P*6_3_/*mmc*) phase, with 54.4% phase fraction. The ε-Fe_3_N type phase is nitrogen-rich, with nitrogen atoms distributed across the three available octahedral sites, as shown in Figure 4A. The N(c) site which is usually used to model stoichiometric ε-Fe_3_N has 77.8% occupancy, N(b) has 46.7% occupancy and only 3.5% of N(d) sites are occupied. This use of three nitrogen sites is consistent with the published Fe_3_N_1.3_ structure ICSD-93175.^26^ The hcp phase has a nitrogen content of 12.3%, consistent with the observation that the metal atoms adopt the hcp arrangement and only a small amount of nitrogen is incorporated into octahedral holes. The published NiAs-type FeN structure with much higher nitrogen content consists of hexagonal close packing of nitrogen and the iron occupying octahedral sites.^27^^,^^28^ Figure 4B gives the perspective view of the unit cell.

**Table 2:** Selected results of Rietveld refinements of PND Data of Ru_1.03_Fe_3_N_1.08_-500°C-24 h

	Space group	Lattice parameters			Phase fraction
		a	c	Volume	
Phase 1	*P*6_3_22	4.77	4.42	87.24	45.6%
Phase 2	*P*6_3_/*mmc*	2.70	4.30	27.14	54.4%

### Ammonia synthesis activity of Ru_x_Fe_3_N_y_

Three typical, representative, samples (Ru_1.03_Fe_3_N_1.08_-500°C-24 h, Ru_0.82_Fe_3_N_0.78_-600°C-12 h, and Ru_0.86_Fe_3_N_0.80_-900°C-12 h) were selected for ammonia synthesis activity testing. The measured ammonia synthesis rates are summarized in Table 3, alongside the nitrogen contents measured from CHN combustion analysis (the expected nitrogen wt. % for RuFe_3_N is 4.95%).

**Table 3:** Ru_x_Fe_3_N_y_ nitrogen content before and after ammonia synthesis reaction and their ammonia synthesis activity at ambient pressure and temperatures between 200°C and 400°C

	Nitrogen content (wt. %)			Ammonia synthesis rate (μmol h^−1^ g^−1^)			
	Pre-catalysis	Post-catalysis		200°C	250°C	300°C	400°C
		200°C-250°C	300°C-400°C				
Ru_1.03_Fe_3_N_1.08_-500°C-24 h	5.27%	0.04%	0.08%	non-steady state	minimal	non-steady state	111 ± 9
Ru_0.82_Fe_3_N_0.78_-600°C-12 h	3.27%	0.14%	0.00%	146	non-steady state	non- steady state	123 ± 33
Ru_0.86_Fe_3_N_0.80_-900°C-12 h	3.81%	2.78%	0.00%	minimal	non-steady state	non-steady state	non-steady state
Fe_2_N	9.87%	–	0.00%	non-steady state	–	non-steady state	56 ± 4

**Figure 5:**
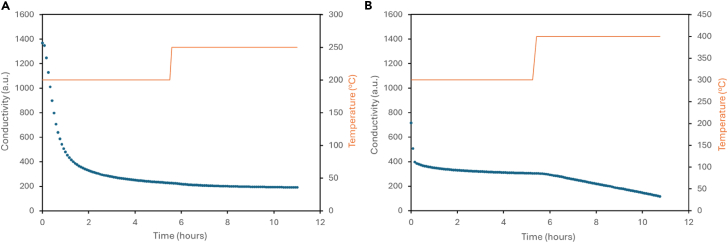
Ammonia synthesis activity of Ru_1.03_Fe_3_N_1.08_-500°C-24 h Conductivity profiles for Ru_1.03_Fe_3_N_1.08_-500°C-24 h reacted with 3:1 H_2_/N_2_ at (A) 200°C and 250°C and (B) 300°C and 400°C. A decrease in conductivity is associated with the formation of ammonia.

The two phase sample Ru_1.03_Fe_3_N_1.08_-500°C-24 h was tested at 200°C for 5 h 30 min, followed by a temperature increase to 250°C, which was maintained for 5 h 25 min. The conductivity profile is shown in Figure 5. In (a), it shows that there was a large decrease in conductivity during the first 2 h of the reaction, indicating that a large amount of ammonia was produced beyond which production of ammonia was minimal with almost no further ammonia being produced after the temperature was increased to 250°C. Upon testing fresh sample at an initial temperature of 300°C non-steady state production of ammonia occurred and upon increasing the reaction temperature further to 400°C, steady state catalytic activity was observed. Extended duration tests (Figure S8) showed steady state ammonia production at a rate of 111 ± 9 μmol h^−1^ g^−1^ for the entire duration of the test (35 h).

**Figure 6:**
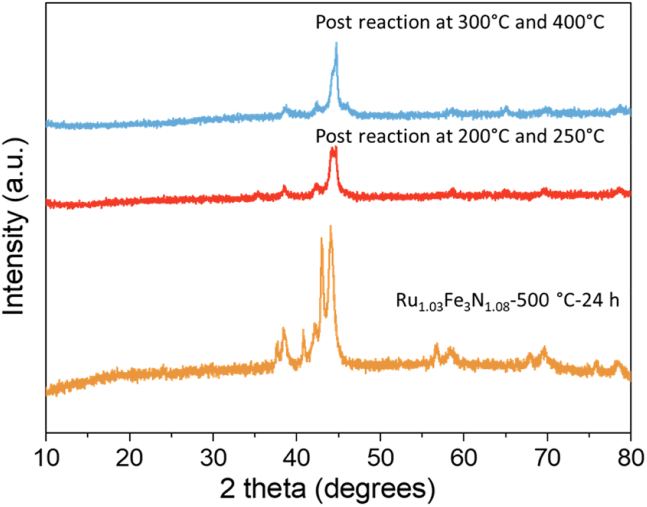
XRD patterns of Ru_1.03_Fe_3_N_1.08_-500°C-24 h before and after ammonia synthesis reaction Pre-reaction, post-reaction at 200°C and 250°C, and post-reaction at 300°C and 400°C.

The pre-reaction XRD pattern of the material is presented in Figure 6 and comprises of the two phases. Upon reaction, with the exception of the reflection at ca 44° 2θ, the peaks associated with the nitride phase are less prominent and they shift to higher 2θ values, which is consistent with the loss of lattice N. Additional reflections at ca. 46° and 65° 2θ are observed in the post-reaction XRD pattern for the longer reaction (Figure S9). The combustion analysis shows there was a reduction in nitrogen content compared to pre-reaction, with almost no nitrogen left after the reaction and the ammonia production observed at both 200°C and 300°C for this material is ascribed to this with the percentage of lattice nitrogen lost from the material being converted to ammonia being close to full conversion (90%). The steady state activity observed at 400°C is ascribed to an iron-ruthenium alloy.

Single-phase sample Ru_0.82_Fe_3_N_0.78_-600°C-12 h displays similar behavior to that of Ru_1.03_Fe_3_N_1.08_-500°C-24 h in ammonia synthesis. The conductivity profiles are shown in Figure S4. The conductivity profile of Ru_0.82_Fe_3_N_0.78_-600°C-12 h shows that there was a large decrease in conductivity during the first hour of the reaction. After 75 min at this temperature, the ammonia production rate was calculated to be 146 μmol h^−1^ g^−1^. The activity was non-steady state after increasing the temperature to 250°C. The post-reaction XRD pattern displayed in Figure S5 reveals that the reflections shifted to higher 2θ values compared to pre-reaction. It is suggested that nitrogen was removed from the material during the reaction and a Fe-Ru alloy was formed. The loss of lattice nitrogen was confirmed through combustion tests. At 300°C, it was noted that the production of ammonia was minimal after the first hour of the reaction and the production of ammonia under these conditions is proposed to be non-catalytic. At 400°C, the activity was found to be catalytic with a rate of 123 ± 33 μmol h^−1^ g^−1^ with steady state activity being observed over 35 h (Figure S8). The Ru-Fe alloy phase was observed in the XRD pattern post-400°C reaction (Figure S10). Compared to the other two samples, single-phase sample Ru_0.86_Fe_3_N_0.80_-900°C-12 h did not generate a great amount of ammonia at 200°C, and was not found to exhibit steady state performance at 400°C (Figures S6 and S7).

**Figure 7:**
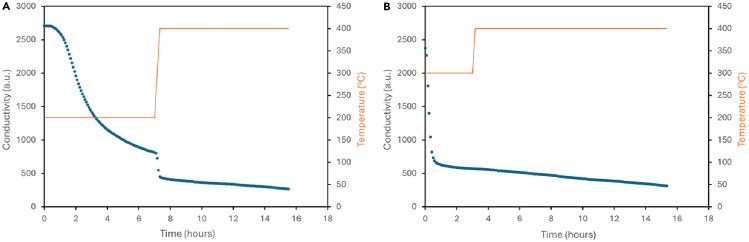
Ammonia synthesis activity of Fe_2_N Conductivity profiles for Fe_2_N reacted with 3:1 H_2_/N_2_ at (A) 200°C and 400°C and (B) 300°C and 400°C. A decrease in conductivity is associated with the formation of ammonia.

As a comparison to the materials investigated in this study, Fe_2_N was prepared and tested. The conductivity profiles are shown in Figure 7. As observed for the other samples, the material has non-steady state activity at both 200°C and 300°C, with the production of ammonia being attributed to the conversion of lattice nitrogen. The post-reaction XRD patterns in Figure S11 and the nitrogen analysis confirms that the iron nitride has been reduced to iron metal during the reaction. At 400°C, the material was observed to have steady state activity over 12 h with a rate of 56 ± 4 μmol h^−1^ g^−1^.

### Computational modeling

Computational modeling has been undertaken with the aim of providing additional insight into the phase stability and reactivity of the Ru-Fe-N system.

**Figure 8:**
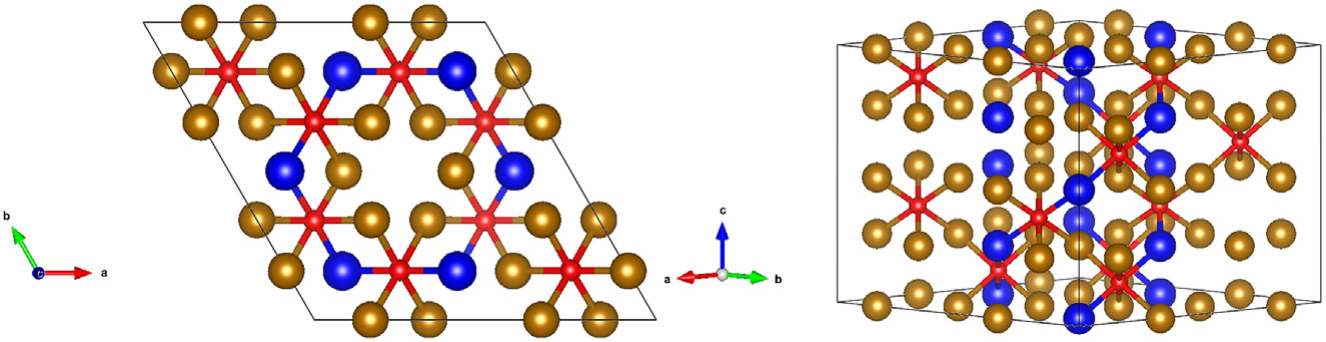
Graphic illustrated the supercell model applied to approximate the disordered nitride phases synthesized, viewing the cell from the top (i.e., along the *c* lattice vector) (left) and from the side (right) Fe atoms: bronze; Ru atoms: blue; N atoms: red.

The disordered phases revealed by the characterization of the synthesized products present a challenge for computational modeling, particularly when employing periodic boundary conditions, as is the case for modeling of bulk materials. Configurational entropy associated with the disordered structure is likely to be an important factor in the greater stability of reported phases, compared to the ordered anti-perovskite phase discussed in previous computational studies. However, valuable insights can still be obtained from approximate models. While multiple disordered phases were identified, it is clear that the phases are highly similar, all essentially consisting of a hcp alloy lattice with varying amounts of interstitial nitrogen. Hence, models for the experimentally reported phases were approximated by constructing a supercell based on the ε-Fe_3_N unit cell (illustrated by Figure 8), with an appropriate fraction of the Fe replaced by Ru, and a fraction of the N removed, to approximate the experimentally determined Ru:Fe:N ratio, with a supercell composition of Ru_12_Fe_36_N_12_. Substituting Ru atoms and N vacancies were distributed evenly throughout the supercell; while the application of periodic boundary conditions to a relatively small supercell necessarily results in a highly ordered system, the model applied nonetheless approximates the local coordination environments and overall stoichiometry. Hence, calculations were performed for the approximate nitride model and its corresponding alloy to assess the energetics of decomposition of the nitride phase and rationalize the experimentally reported difficulty in re-nitriding the samples (see in the futher section). Furthermore, calculations were performed for a model cubic anti-perovskite of the same stoichiometry, in order to assess the relative stability of the two phases.

The calculated lattice parameters for the disordered hcp nitride phase and hypothetical cubic anti-perovskite phase are summarized in Table 4. For the disordered hcp nitride phase, while a direct comparison with the experimentally prepared samples is challenging due to the disorder and variation in composition, the optimized supercell lattice vectors are broadly in agreement with the experimentally determined lattice vectors. For the model based on the 2 × 2 × 2 ε-Fe_3_N supercell employed, optimized lattice parameters of *a* = 9.038 Å; *c* = 8.409 Å were obtained, which corresponds to *a* = 4.519 Å; *c* = 4.214 Å. Compared with the experimentally determined values of *a* = 4.77 Å; *c* = 4.42 Å (Table 2), the DFT-optimized values are within 5.26% and 4.88% for the *a* and *c* lattice parameters, respectively. The application of the D3 dispersion correction scheme and thermal expansion are likely the reasons for the slight underestimation of the lattice parameters as determined from density functional theory (DFT) calculations. For the hypothetical cubic anti-perovskite phase, the DFT-optimized lattice parameter of 3.63 Å is corroborated by previous computational studies,^23^^,^^24^ although a comparison with experimentally determined values is not possible due to the paucity of evidence for its existence in the experimental literature, including the inability to prepare it in the present study.

**Table 4:** Calculated decomposition energies, cell lattice vectors and relative stabilities for the disordered hexagonal model phase and the cubic anti-perovskite model phase, along with the calculated relative stability of the two nitride phases (with respect to the more stable structure)

Model Composition	Lattice parameters/Å	*E*_decomp._ (N_2_)/eV Per N atom	*E*_decomp._ (NH_3_)/eV Per N atom	Relative stability per Ru atom/eV
Disordered nitride: Ru_12_Fe_36_N_12_	*a* = 9.038; *c* = 8.409	−0.980	−2.068	0.000
Anti-perovskite: RuFe_3_N	*a* = 3.630	−0.825	−1.192	+0.355

The calculated bulk decomposition energies for the model disordered nitride to its corresponding alloy are summarized in Table 4, along with the corresponding values for the cubic anti-perovskite, and the relative stabilities of the two model phases. The calculations show that for both systems, decomposition of the nitride to its corresponding alloy is exothermic, both under reducing conditions (i.e., via hydrogenation to yield ammonia from lattice N) and with respect to loss of lattice N to gaseous N_2_. This reflects experimentally observed loss of lattice N to yield ammonia under ammonia synthesis conditions, and the reported difficulty in re-nitriding the resulting alloy (see in the further section), since the process is therefore endothermic.

The calculated relative stability also reveals that the model disordered hcp nitride is more stable than the corresponding cubic perovskite, corroborating the experimental results that suggest that no anti-perovskite phase is formed. It must also be noted that while the DFT-calculated relative stabilities can provide valuable insights into which phases are likely to be formed, contributions from configurational entropy have been neglected and are likely to play a role in the stability of the disordered phase, thus enhancing its stability compared to the ordered cubic perovskite phase.

Furthermore, it can be seen that the calculated decomposition energies for the model anti-perovskite phase are less endothermic compared to those for the disordered hexagonal phase; this implies that the resulting cubic alloy from anti-perovskite decomposition is also less stable than the hexagonal alloy resulting from decomposition of the disordered phase; indeed, the calculations show that the cubic alloy is less stable than the hexagonal alloy by +0.511 eV per Ru atom.

In summary, the DFT calculations corroborate the experimentally observed absence of a cubic anti-perovskite phase, and the facile loss of lattice N. It therefore appears likely that nitrogen chemical looping is unfeasible for the RuFe_3_N system, and that the experimentally reported catalytic activity can be attributed to the emergence of a Ru-Fe alloy phase resulting from the loss of lattice N from the nitride. Such a system has already been subject to a DFT surface activity study, which revealed that d-band modifications due to Ru-Fe alloying lead the emergence of enhanced ammonia synthesis activity.^29^

### Regeneration

**Figure 9:**
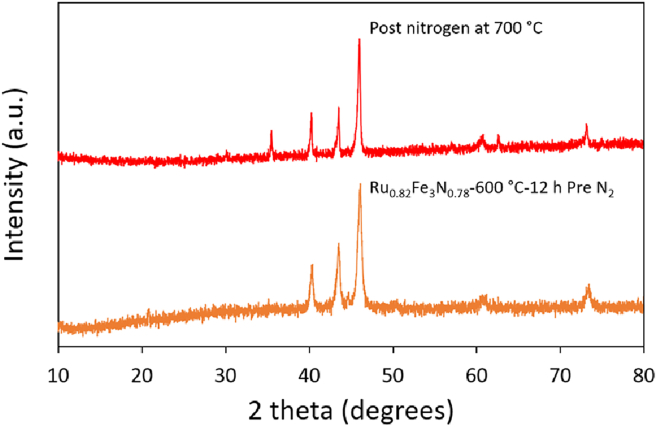
XRD patterns of Ru_0.82_Fe_3_N_0.78_-600°C-12 h pre- and post-treatment with nitrogen Pre-nitrogen and post-nitrogen at 700°C.

The close to full conversion of lattice nitrogen to ammonia upon reaction at lower temperatures for some of the Ru-Fe systems renders them of possible interest as nitrogen looping agents for ammonia synthesis. In order to be viable, re-nitridation of N-depleted phases with N_2_ would be highly desirable. Accordingly re-nitridation of reduced phases has been investigated. To regenerate Ru_0.82_Fe_3_N_0.78_-600°C-12 h and Ru_0.86_Fe_3_N_0.80_-900°C-12 h from the Ru-Fe alloy, the post-reaction materials were treated with nitrogen at 700°C for 4 h. The XRD patterns in Figure 9 and nitrogen analysis show that the Ru_0.82_Fe_3_N_0.78_-600°C-12 h nitride had not been regenerated under these conditions. An additional reflection due to iron oxide at ca. 35° 2θ post-nitrogen treatment suggests that phase separation has occurred with oxidation happening upon discharge of the sample into air prior to the diffraction measurement. A similar result was obtained when attempting to regenerate Ru_0.86_Fe_3_N_0.80_-900°C-12 h from Ru-Fe alloy (Figure S12).

It was also of interest to check whether re-nitridation of a partially N-depleted system was possible. Accordingly, Ru_1.03_Fe_3_N_1.08_-500°C-24 h was tested at 200°C for only 30 min to partially remove some of the lattice nitrogen. This was performed in order to retain the original structure of the nitride and potentially assist in the re-nitridation of the material. After losing nitrogen from ammonia synthesis, the post-reaction Ru_1.03_Fe_3_N_1.08_-500°C-24 h was then treated with nitrogen at 700°C for 4 h. The XRD pattern of the material post-treatment shows that the nitride has transformed to the alloy phase (Figure S13). The Fe_3_O_4_ phase emerged after nitrogen treatment. From the nitrogen analysis in Table S7, it can be seen that the lattice nitrogen has been completely removed from this material after nitridation treatment. Figure S14 gives the SEM images of sample Ru_1.03_Fe_3_N_1.08_-500°C-24 h before and after ammonia synthesis reaction and after nitrogen regeneration, and sample Ru_0.82_Fe_3_N_0.78_-600°C-12 h before and after reaction. The EDX compositional analysis is provided in Table S8. SEM images show that the surface morphologies were unchanged after ammonia synthesis reactions, even though the lattice nitrogen was removed from these samples during the reaction. EDX shows the Ru and Fe ratios are similar before and after reaction. After N_2_ regeneration, SEM image shows that Ru_1.03_Fe_3_N_1.08_-500°C-24 h has a slightly smoother surface, which is possibly caused by the sample transforming to the alloy phase after regeneration, which has been proved by XRD. We conclude that—despite its high selectivity to NH_3_ upon low temperature reduction—the Ru_x_Fe_3_N_y_ system is likely to be an unsuitable candidate material for chemical looping.

## Discussion

A series of ruthenium iron nitride phases with Ru:Fe around 1:3 were synthesized using a citrate-gel method followed by ammonolysis. The iron and ruthenium ratio, ammonolysis temperature and duration were varied, the structures and catalytic performances in ammonia synthesis were investigated. When ammonolysis temperature is higher than 500°C, XRD shows that Ru_x_Fe_3_N_y_ has ε-Fe_3_N *P*6_3_22 structure, while they have two phases when ammonolysis temperature is under 500°C. As a typical two-phase sample, PND was conducted on sample Ru_1.03_Fe_3_N_1.08_-500°C-24 h. Rietveld refinement shows that the model of a ε-Fe_3_N (*P*6_3_22) phase and a phase with hexagonal close packed metal atoms and disordered nitrogen (model based on NiAs in *P*6_3_/*mmc* but with cation and anion positions reversed) can fit well with Ru_1.03_Fe_3_N_1.08_-500°C-24 h PND pattern. The ε-Fe_3_N type phase is nitrogen-rich and the hcp phase has low nitrogen content. CHN elemental analysis shows that low ammonolysis temperature (400°C–500°C) contributes to high nitrogen content in the materials while high ammonolysis temperature (900°C) leads to low nitrogen content. Nitrogen content evaluated by CHN analysis was 5.78 wt. % for ammonolysis at 400°C and 2.69 wt. % ammonolysis at 900°C, the theoretical value is 4.94 wt. % for RuFe_3_N. Steady state catalytic activity for ammonia synthesis is observed for these systems at 400°C and has been associated with the presence of Ru-Fe alloy. Despite the promising high selectivity of conversion of lattice N to NH_3_ at lower temperatures (200°C–300°C), the inability to regenerate the N depleted phases with N_2_ limits the interest in them as looping agents. DFT calculations support the experimental findings, revealing that the disordered hcp metal nitride system is likely to be more stable than any isomeric cubic anti-perovskite phase, and that the nitride phase is likely to readily undergo decomposition, with loss of lattice N to yield the corresponding alloy being exothermic, explaining the difficulty in regenerating the nitride and thus limiting scope for chemical looping behavior. The observed catalytic activity can be attributed to the resulting alloy facilitating ammonia synthesis, and is consistent with previous computational studies.

### Limitations of the study

A variety of models have been used to fit the neutron diffraction data. The model of a ε-Fe_3_N (*P*6_3_22) phase and a phase with hexagonal close packed metal atoms and disordered nitrogen provided a better fit to the data. The limitation is the data fitting is still not perfect, the intensities of a few peaks do not perfectly fit. In addition, the complicated phases of the synthesized products are also a challenge for the computational modeling.

## Resource availability

### Lead contact

Further information and requests for resources and reagents should be directed to and will be fulfilled by the lead contact, Justin Hargreaves (justin.hargreaves@glasgow.ac.uk).

### Materials availability

This study did not generate new unique reagents.

### Data and code availability

• All data reported in this paper will be shared by the lead contact upon request.
• This paper does not report original code.
• Any additional information required to reanalyse the data reported in this paper is available from the lead contact upon request.

## Acknowledgments

The authors wish to acknowledge the 10.13039/501100000266EPSRC for the research grants EP/T027851/1, EP/T028416/1, and EP/T028629/1. We also thank ISIS for the Polaris beam time under grant XB2291110. The authors acknowledge the UK Catalysis Hub Consortium (funded by 10.13039/501100000266EPSRC under EP/R026815/1) for the provision of additional resources. M.D.H. acknowledges the STFC Scientific Computing Department’s SCARF cluster, and the ARCHER2 UK National Supercomputing Service (http://www.archer2.ac.uk), for the provision of computational resources, the latter enabled via membership of the Materials Chemistry Consortium, funded by 10.13039/501100000266EPSRC (EP/X035859).

## Author contributions

Conceptualization, C.R.A.C., J.S.J.H., and A.L.H.; methodology, L.S., A.D., M.H., C.R.A.C., J.S.J.H., and A.L.H.; software, M.H. and C.R.A.C.; investigation, L.S., A.D., and M.H.; writing—original draft, L.S., A.D., M.H., and J.S.J.H.; writing—review and editing, L.S., A.D., M.H., C.R.A.C., J.S.J.H., and A.L.H.; supervision, C.R.A.C., J.S.J.H., and A.L.H.; funding acquisition, C.R.A.C., J.S.J.H., and A.L.H.

## Declaration of interests

The authors declare no competing interests.

## STAR★Methods

### Key resources table

**Table:** 

REAGENT or RESOURCE	SOURCE	IDENTIFIER
**Chemicals, peptides, and recombinant proteins**		

Ruthenium trichloride (RuCl_3_, (Ru content 45-55%))	Sigma-Aldrich	208523
Iron (III) nitrate nonahydrate (>98%)	Sigma-Aldrich	216828
Sulfuric Acid (99.999%)	Sigma-Aldrich	339741
Citric acid monohydrate (99.5%)	Thermofisher Scientific	5949-29-1
Nitric acid (70%)	Thermofisher Scientific	7697-37-2
Ammonia (anhydrous grade)	BOC	07664-41-7
75% H_2_/N_2_ (H2: 99.998%, N2: 99.995%)	BOC	155367-L-C

**Software and algorithms**		

VASP 5.4.4	Kresse et al.^30^^,^^31^^,^^32^^,^^33^	https://www.vasp.at/
ICSD	PSDS	https://www.psds.ac.uk/icsd
GSAS-2	GSAS-II	https://advancedphotonsource.github.io/GSAS-II-tutorials/

**Other**		

Bruker D2 Phaser X-ray diffractometer	Bruker	https://www.bruker.com/en/products-and-solutions/diffractometers-and-x-ray-microscopes/x-ray-diffractometers/d2-phaser.html
ZEISS Sigma 500 VP FE-SEM	ZEISS	https://www.zeiss.com/microscopy/en/products/sem-fib-sem/sem.html
POLARIS diffractometer	ISIS	https://www.isis.stfc.ac.uk/Pages/polaris.aspx

### Method details

#### Synthesis of ruthenium iron nitrides

Ruthenium iron nitride compounds with a target composition of 1:3:1 Ru:Fe:N were fabricated by a citrate-gel method followed by ammonolysis. 0.6-1.6 mmol ruthenium(III) chloride, 3 mmol iron(III) nitrate nonahydrate and 40 mmol citric acid monohydrate were dissolved in 60 mL 2.6 mol dm^-3^ nitric acid. For example, for the solution with molar ratio of Ru:Fe=1.4:3, 0.2904 g ruthenium(III) chloride, 1.2120 g iron(III) nitrate nonahydrate and 8.4056 g citric acid monohydrate were dissolved in 60 mL 2.6 mol dm^-3^ nitric acid. The dark red solution was evaporated in a sand bath at 90°C for ∼20 h to obtain a red gel. *Warning*: the sand bath was used because citrate gels can occasionally ignite. The gel was heated in an ashing furnace (60°C min^−1^ heating rate) at 500°C for 2 h. The obtained greyish red foam was ground to a powder then heated in flowing ammonia (BOC anhydrous grade, further dried with molecular sieves) to 400-900°C at 5 °C min^−1^ and the temperature was maintained for 12-168 h. This process is called ammonolysis. Once cooled to room temperature, the furnace tube was flushed with N_2_ for 60 min, followed by allowing air to diffuse into the tube slowly (*Warning*: unpassivated metal nitrides are pyrophoric).

#### Catalytic performance of ruthenium iron nitride catalysts in ammonia synthesis

The catalytic performance of ruthenium iron nitrides in ammonia synthesis were evaluated at atmospheric pressure under a 3:1 ratio of H_2_/N_2_ (BOC, H_2_: 99.998%, N_2_: 99.995%) with a gas flow rate of 60 cm^3^ min^-1^. The tested sample was loaded into a silica tube and placed in the furnace. The furnace was heated to the required temperature at a rate of 10°C min^-1^. The materials were tested at a temperature of between 200-400°C. The mixed H_2_/N_2_ gas passed through a dilute sulfuric acid solution (0.00108 mol dm^-3^) and the conductivity values were recorded every five minutes with a HACH HQ14d portable conductivity meter. The generated ammonia reacted with the sulfuric acid and changed its conductivity. Ammonia production was calculated from the conductivity decrease of the sulfuric acid solution with time. Accordingly, the conductivity plots presented in this study correspond to those of the dilute sulfuric acid solution with arbitrary units corresponding to those instances where the replenishment of the standard solution was necessary.

#### Characterisation

The powder X-ray diffraction (PXRD) patterns were collected with a Bruker D2 Phaser X-ray diffractometer with Cu-K_α_ radiation. The Rigaku PDXL2 package and ICSD were used for diffraction pattern matching. Rietveld refinement was conducted with the GSAS-2 package. Combustion (CHN) analysis was conducted by Medac Ltd. Scanning electron microscopy (SEM) and energy-dispersive X-ray (EDX) spectra and mapping were collected with a ZEISS Sigma 500 VP FE-SEM. Powder neutron diffraction (PND) was performed at the high-intensity time-of-flight POLARIS diffractometer at the ISIS source. Diffraction data were recorded on three different detector banks located at about 2θ = 35, 90, and 135° which cover different ranges of d spacings.

#### Computational details

In order to assess the stability of the synthesied phase and rationalise the observed loss of lattice nitrogen, plane-wave Density Functional Theory (DFT) as implemented in the VASP code (v.5.4.4)^30^^,^^31^^,^^32^^,^^33^ was applied to bulk models approximating the experimentally determined structures, and for the isomeric hypothetical cubic anti-perovskite phase. For the experimentally reported disordered hexagonal nitrides, a supercell approach based on the ε-Fe_3_N unit cell was employed, with a 2x2x2 supercell being constructed. A quarter of the Fe atoms were replaced with Ru, and a quarter of the N atoms removed, corresponding to a 1:3:1 Ru:Fe:N ratio which aligns with the experimentally determined compositions. A model for the corresponding alloy phase was constructed by removing the remaining N atoms. All cell lattice vectors and atomic coordinates were relaxed to within 0.01 eVÅ^-1^. The revised Perdew-Burke-Ernzerhof (RPBE) exchange correlation functional was used throughout,^34^ with the D3 dispersion correction with Becke-Johnson damping applied.^35^^,^^36^ A Monkhorst–Pack k-point sampling scheme^37^ was used with a k-point mesh of 4x4x4, commensurate with the cell dimensions. For the cubic anti-perovskite model (and its corresponding alloy), a k-point sampling mesh of 7x7x7 was implemented. Inner electrons were replaced by projector augmented waves (PAW),^38^ and the valence states were expanded in plane-waves with a cut-off energy of 600 eV.

## References

[1] Pfromm P.H. (2017). Towards sustainable agriculture: Fossil-free ammonia. J. Renew. Sustain. Energy.

[2] Ye T.-N., Park S.-W., Lu Y., Li J., Sasase M., Kitano M., Hosono H. (2020). Contribution of Nitrogen Vacancies to Ammonia Synthesis over Metal Nitride Catalysts. J. Am. Chem. Soc..

[3] Nishibayashi Y. (2015). Recent Progress in Transition-Metal-Catalyzed Reduction of Molecular Dinitrogen under Ambient Reaction Conditions. Inorg. Chem..

[4] Humphreys J., Lan R., Tao S. (2021). Development and Recent Progress on Ammonia Synthesis Catalysts for Haber–Bosch Process. Adv. Energy Sustain. Res..

[5] Jacobsen C.J., Dahl S., Clausen B.S., Bahn S., Logadottir A., Nørskov J.K. (2001). Catalyst Design by Interpolation in the Periodic Table: Bimetallic Ammonia Synthesis Catalysts. J. Am. Chem. Soc..

[6] Kojima R., Aika K.-I. (2001). Cobalt molybdenum bimetallic nitride catalysts for ammonia synthesis: Part 1. Preparation and characterization. Appl. Catal., A.

[7] Kojima R., Aika K.-I. (2001). Cobalt molybdenum bimetallic nitride catalysts for ammonia synthesis: Part 2. Kinetic study. Appl. Catal., A.

[8] Kojima R., Aika K.-I. (2001). Cobalt molybdenum bimetallic nitride catalysts for ammonia synthesis: Part 3. Reactant gas treatment. Appl. Catal., A.

[9] Kojima R., Aika K.-I. (2003). Cobalt Molybdenum Bimetallic Nitride Catalysts for Ammonia Synthesis. Chem. Lett..

[10] Daisley A., Hargreaves J.S.J. (2023). Metal nitrides, the Mars-van Krevelen mechanism and heterogeneously catalysed ammonia synthesis. Catal. Today.

[11] Zeinalipour-Yazdi C.D., Hargreaves J.S.J., Catlow C.R.A. (2016). DFT-D3 Study of Molecular N2 and H2 Activation on Co3Mo3N Surfaces. J. Phys. Chem. C.

[12] Zeinalipour-Yazdi C.D., Hargreaves J.S.J., Catlow C.R.A. (2015). Nitrogen Activation in a Mars–van Krevelen Mechanism for Ammonia Synthesis on Co3Mo3N. J. Phys. Chem. C.

[13] Zeinalipour-Yazdi C.D., Hargreaves J.S.J., Catlow C.R.A. (2018). Low-T Mechanisms of Ammonia Synthesis on Co3Mo3N. J. Phys. Chem. C.

[14] Al Sobhi S., Bion N., Hargreaves J.S., Hector A.L., Laassiri S., Levason W., Lodge A.W., McFarlane A.R., Ritter C. (2019). The reactivity of lattice nitrogen within the Ni2Mo3N and NiCoMo3N phases. Mater. Res. Bull..

[15] Bion N., Can F., Cook J., Hargreaves J.S.J., Hector A.L., Levason W., McFarlane A.R., Richard M., Sardar K. (2015). The role of preparation route upon the ambient pressure ammonia synthesis activity of Ni2Mo3N. Appl. Catal., A.

[16] Al Sobhi S., Hargreaves J.S.J., Hector A.L., Laassiri S. (2019). Citrate-gel preparation and ammonia synthesis activity of compounds in the quaternary (Ni,M)2Mo3N (M = Cu or Fe) systems. Dalton Trans..

[17] Goto Y., Daisley A., Hargreaves J.S.J. (2021). Towards anti-perovskite nitrides as potential nitrogen storage materials for chemical looping ammonia production: Reduction of Co3ZnN, Ni3ZnN, Co3InN and Ni3InN under hydrogen. Catal. Today.

[18] Daisley A., Higham M., Catlow C.R.A., Hargreaves J.S.J. (2023). Experimental and theoretical investigations on the anti-perovskite nitrides Co3CuN, Ni3CuN and Co3MoN for ammonia synthesis. Faraday Discuss.

[19] Brown D.E., Edmonds T., Joyner R.W., McCarroll J.J., Tennison S.R. (2014). The Genesis and Development of the Commercial BP Doubly Promoted Catalyst for Ammonia Synthesis. Catal. Lett..

[20] Puvaneswari S., Priyanga G.S., Rajeswarapalanichamy R., Santhosh M. (2015). Structural, electronic, elastic and magnetic properties of RuFe3N and OsFe3N: A first principle study. AIP Conf. Proc..

[21] Paduani C. (2004). Electronic structure of the perovskite-type nitride RuFe3N. J. Magn. Magn Mater..

[22] dos Santos A.V., Kuhnen C.A. (2009). Electronic structure and magnetic properties of RuFe3N nitride. J. Solid State Chem..

[23] Zhao E., Xiang H., Meng J., Wu Z. (2007). First-principles investigation on the elastic, magnetic and electronic properties of MFe3N (M=Fe, Ru, Os). Chem. Phys. Lett..

[24] Hocine K., Rabah M., Rached D., Djili S., Baltache H. (2012). Ab initio study of electronic structure and magnetic properties of MFe3N (M=Ru and Os). Comput. Mater. Sci..

[25] Leineweber A., Jacobs H., Hull S. (2001). Ordering of Nitrogen in Nickel Nitride Ni3N Determined by Neutron Diffraction. Inorg. Chem..

[26] Leineweber A., Jacobs H., Hüning F., Lueken H., Kockelmann W. (2001). Nitrogen ordering and ferromagnetic properties of ϵ-Fe3N1+x (0.10≤x≤0.39) and ϵ-Fe3(N0.80C0.20)1.38. J. Alloys Compd..

[27] Clark W.P., Steinberg S., Dronskowski R., McCammon C., Kupenko I., Bykov M., Dubrovinsky L., Akselrud L.G., Schwarz U., Niewa R. (2017). High-Pressure NiAs-Type Modification of FeN. Angew. Chem. Int. Ed..

[28] Bykov M., Bykova E., Aprilis G., Glazyrin K., Koemets E., Chuvashova I., Kupenko I., McCammon C., Mezouar M., Prakapenka V. (2018). Fe-N system at high pressure reveals a compound featuring polymeric nitrogen chains. Nat. Commun..

[29] Ghuman K.K., Tozaki K., Sadakiyo M., Kitano S., Oyabe T., Yamauchi M. (2019). Tailoring widely used ammonia synthesis catalysts for H and N poisoning resistance. Phys. Chem. Chem. Phys..

[30] Kresse G., Hafner J. (1993). Ab initio molecular dynamics for liquid metals. Phys. Rev. B.

[31] Kresse G., Hafner J. (1994). Ab initio molecular-dynamics simulation of the liquid-metal-amorphous- semiconductor transition in germanium. Phys. Rev. B.

[32] Kresse G., Furthmüller J. (1996). Efficiency of ab-initio total energy calculations for metals and semiconductors using a plane-wave basis set. Comput. Mater. Sci..

[33] Kresse G., Furthmüller J. (1996). Efficient iterative schemes for ab initio total-energy calculations using a plane-wave basis set. Phys. Rev. B.

[34] Hammer B., Hansen L.B., Nørskov J.K. (1999). Improved adsorption energetics within density-functional theory using revised Perdew-Burke-Ernzerhof functionals. Phys. Rev. B.

[35] Grimme S., Antony J., Ehrlich S., Krieg H. (2010). A consistent and accurate ab initio parametrization of density functional dispersion correction (DFT-D) for the 94 elements H-Pu. J. Chem. Phys..

[36] Grimme S., Ehrlich S., Goerigk L. (2011). Effect of the damping function in dispersion corrected density functional theory. J. Comput. Chem..

[37] Monkhorst H.J., Pack J.D. (1976). Special points for Brillouin-zone integrations. Phys. Rev. B.

[38] Blöchl P.E. (1994). Projector augmented-wave method. Phys. Rev. B.

