# Supplementary material for: Structures and ammonia synthesis activity of hexagonal ruthenium iron nitride phases
Source: iScience. 2024 Aug 23;27(9):110795. doi: 10.1016/j.isci.2024.110795 (PMC11406097; doi:10.1016/j.isci.2024.110795)
Supplement: Document S1. Figures S1–S14 and Tables S1–S8 [file mmc1.pdf]

iScience, Volume *27*

Supplemental information

Structures and ammonia synthesis activity

# of hexagonal ruthenium iron nitride phases

Li Shao, Angela Daisley, Michael Higham, C. Richard A. Catlow, Justin S.J. Hargreaves, and Andrew L. Hector

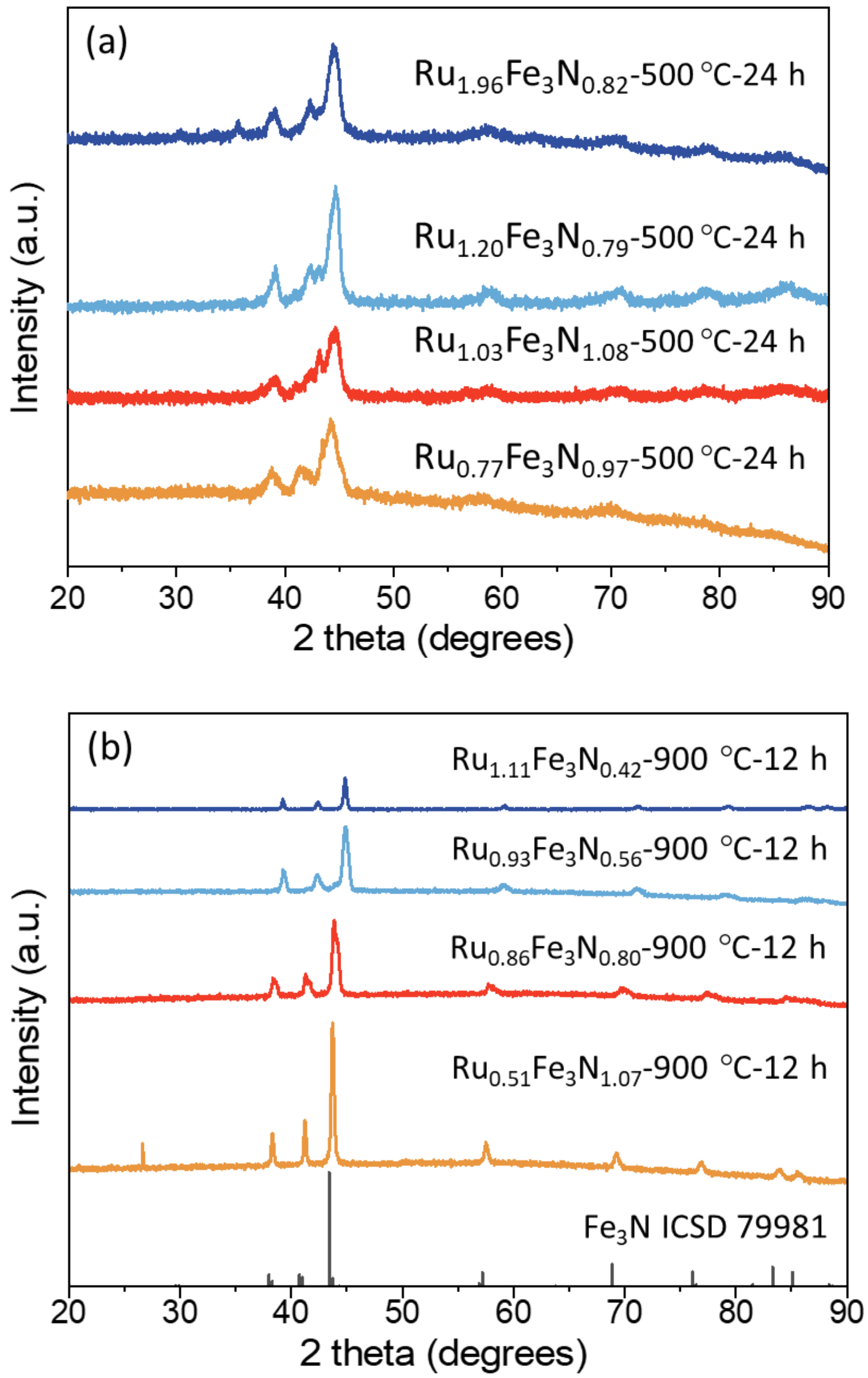

Figure S1: XRD patterns of the $Ru_xFe_3N_y$ samples prepared from the solution with different ruthenium and iron molar ratios, related to Table 1. A.) at ammonolysis temperature of 500 °C; the patterns were obtained from scanning for 24 hours. B.) and ammonolysis temperature of 900 °C; the patterns were obtained from scanning for 2 hours.

The ammonolysis durations were also controlled as 12 h, 24 h and 168 h; the XRD patterns are shown in Figure S2. The ruthenium and iron molar ratio in the precursor solution is 1.4:3. The ammonolysis temperature was 500 °C. Table S4 presents the lattice parameters and nitrogen content of the samples. When ammonolysis is increased from 12 hours to 24 hours, the elemental analysis shows that slightly more nitrogen can be accommodated into the final structure, and lattice parameters of the crystal structure therefore increase. For longer ammonolysis durations of over 168 h, $Ru_{1.15}Fe_3N_{0.82}$-500 °C-168 h starts losing nitrogen, and lattice parameters decrease.

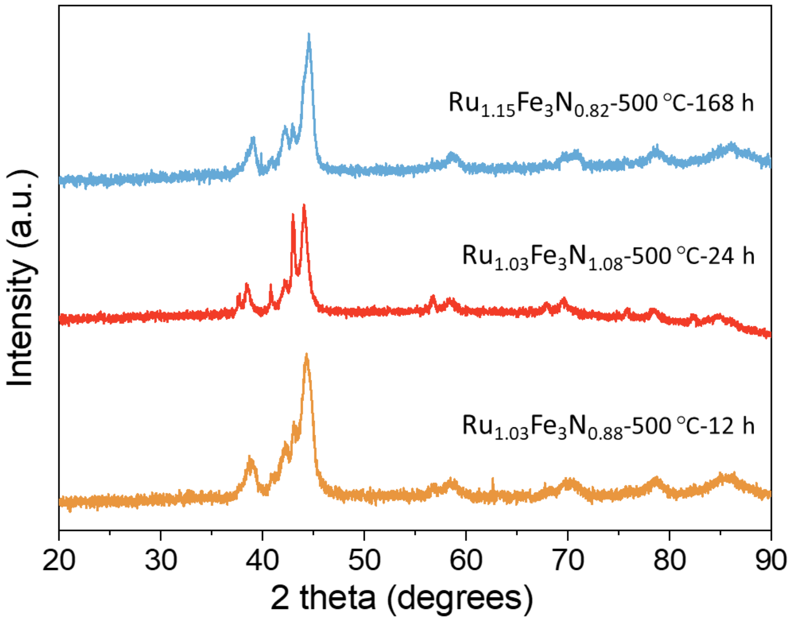

Figure S2: XRD patterns of $Ru_xFe_3N_y$ samples prepared under different ammonolysis durations ranging from 12 hours to 168 hours, related to Table 1.

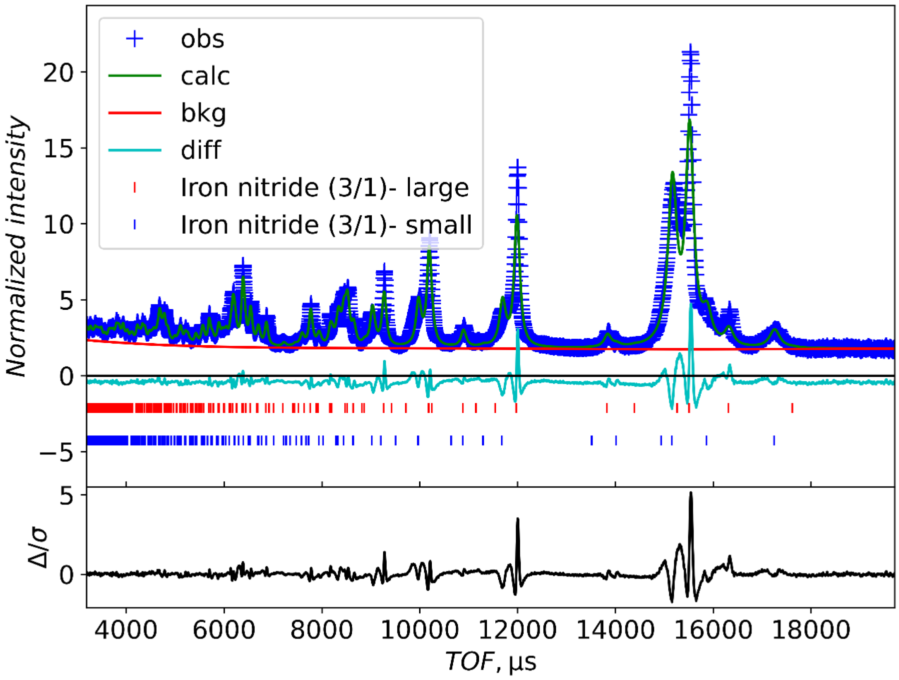

Figure S3: PND pattern of $Ru_{1.03}Fe_3N_{1.08}$-500 °C-24 h fitted with two ε-Fe_3N type $P6_322$ phases, related to Figure 3.

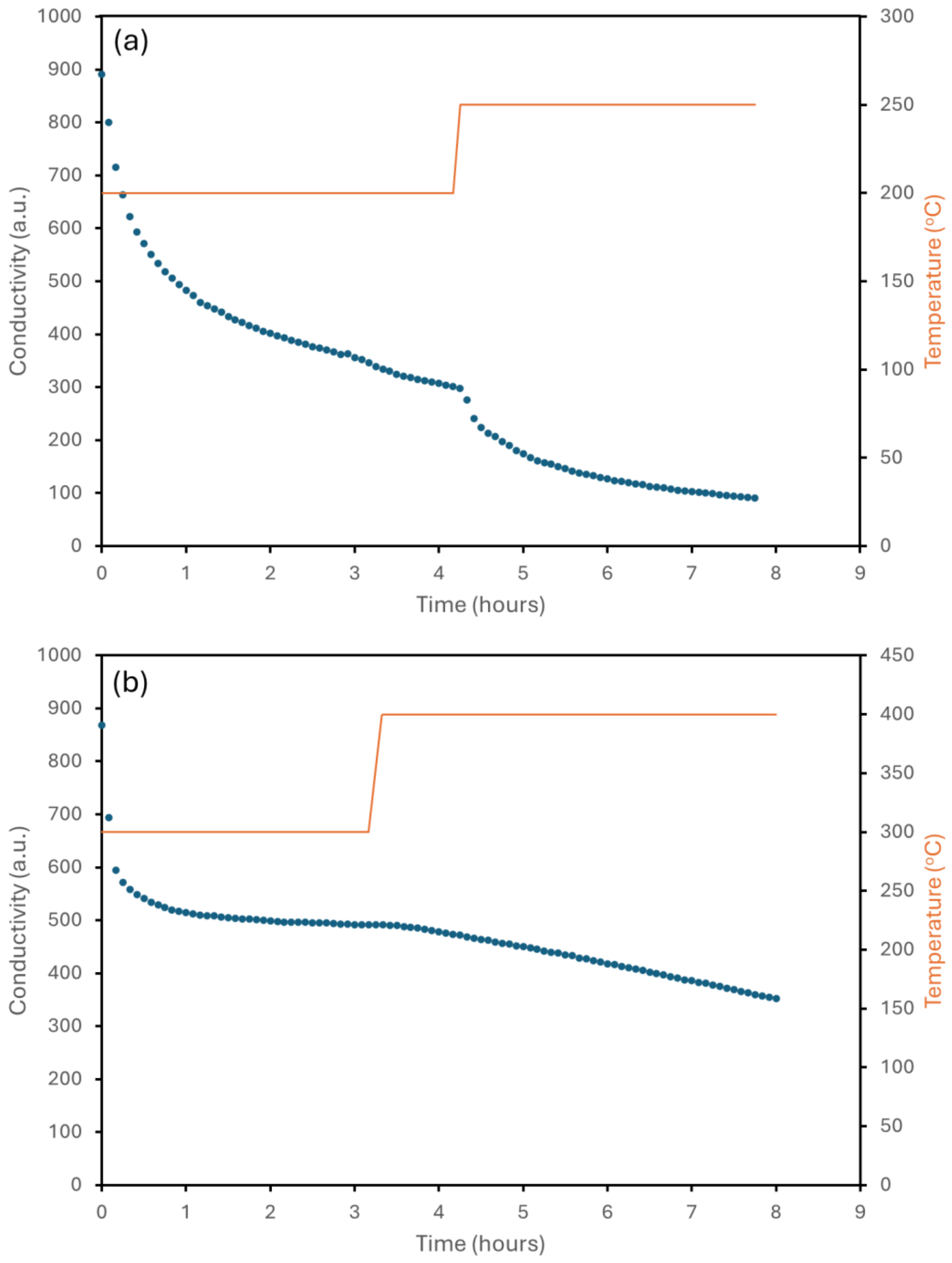

Figure S4: Conductivity profiles for $Ru_{0.82}Fe_3N_{0.78}$-600 °C-12 h reacted with 3:1 H_2/N_2, related to Table 3. A.) 200 °C for 4 h 10 min and then, 250 °C for 3 h 30 min. B.) 300 °C for 3 h 10 min and then, 400 °C for 4 h 40 min. The conductivity relates to that of the standard dilute sulfuric acid solution through which the reactor effluent is flowed. Decreasing conductivity is associated with the formation of ammonia.

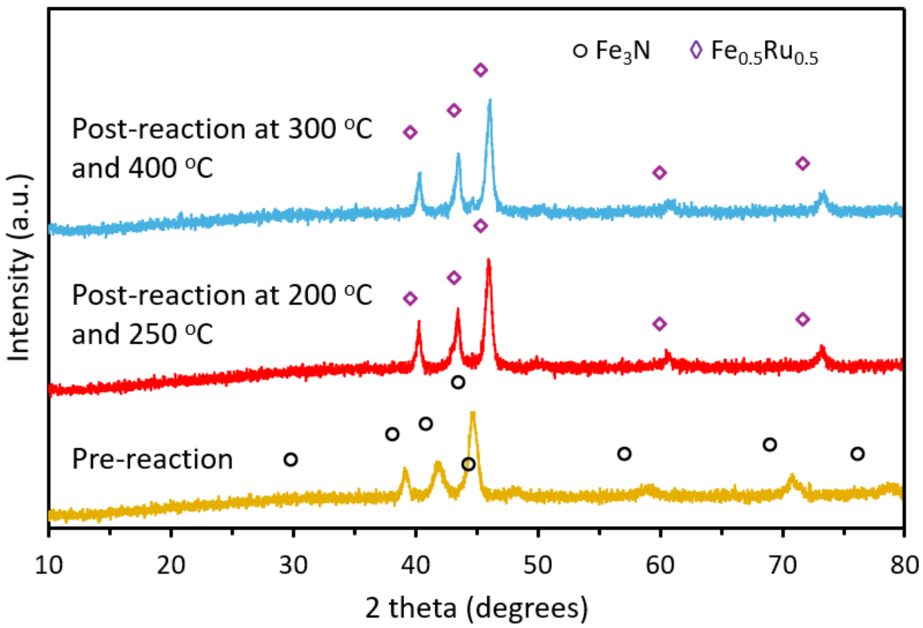

Figure S5: Comparison of XRD patterns for $Ru_{0.82}Fe_3N_{0.78}$-600 ℃ -12 h pre- and post-reaction with 3:1 H_2/N_2, related to Figure 6. A.) pre-reaction. B.) post-reaction at 200 ℃ and 250 ℃. C.) post-reaction at 300 ℃ and 400 ℃.

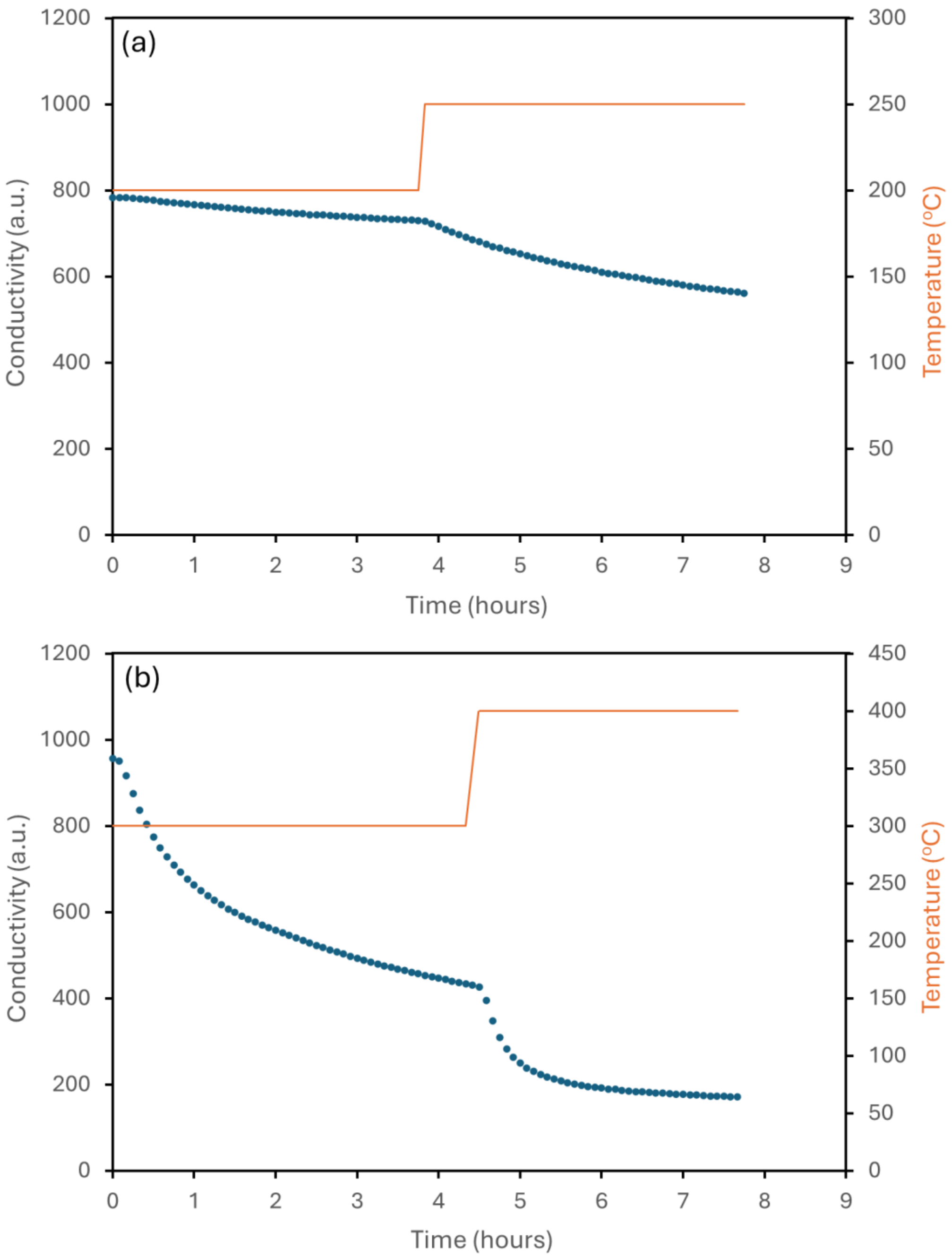

Figure S6: Conductivity profiles for $Ru_{0.86}Fe_3N_{0.80}$-900 °C -12 h reacted with 3:1 H_2/N_2, related to Table 3. A.) 200 °C for 3 h 45 min and then, 250 °C for 3 h 55 min. B.) 300 °C for 4 h 20 min and then, 400 °C for 3 h 10 min.

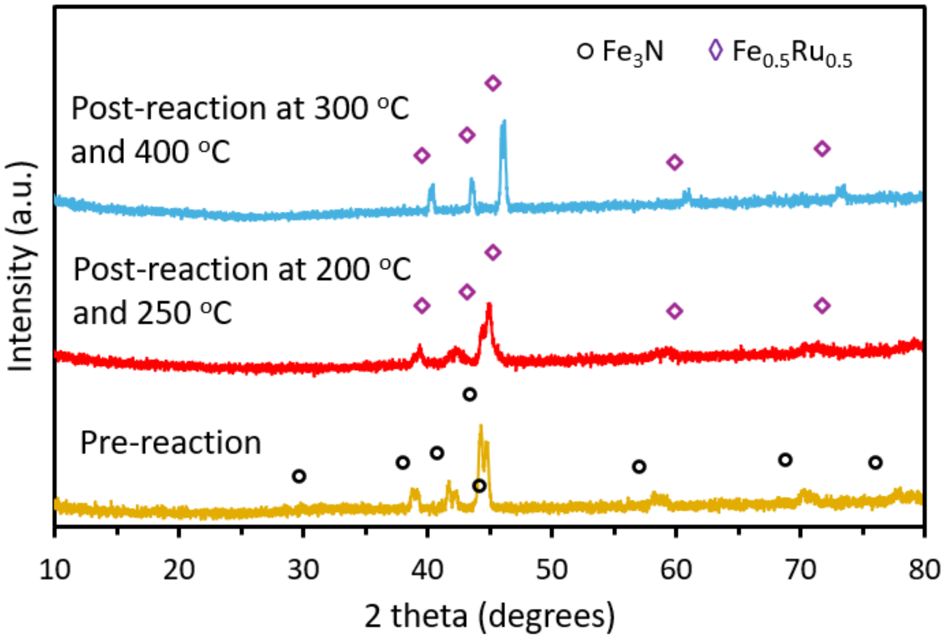

Figure S7: Comparison of XRD patterns for $Ru_{0.86}Fe_3N_{0.80}$-900 °C-12 h pre- and post-reaction with 3:1 H_2/N_2, related to Figure 6. A.) pre-reaction. B.) post-reaction at 200 °C and 250 °C. C.) post-reaction at 300 °C and 400 °C.

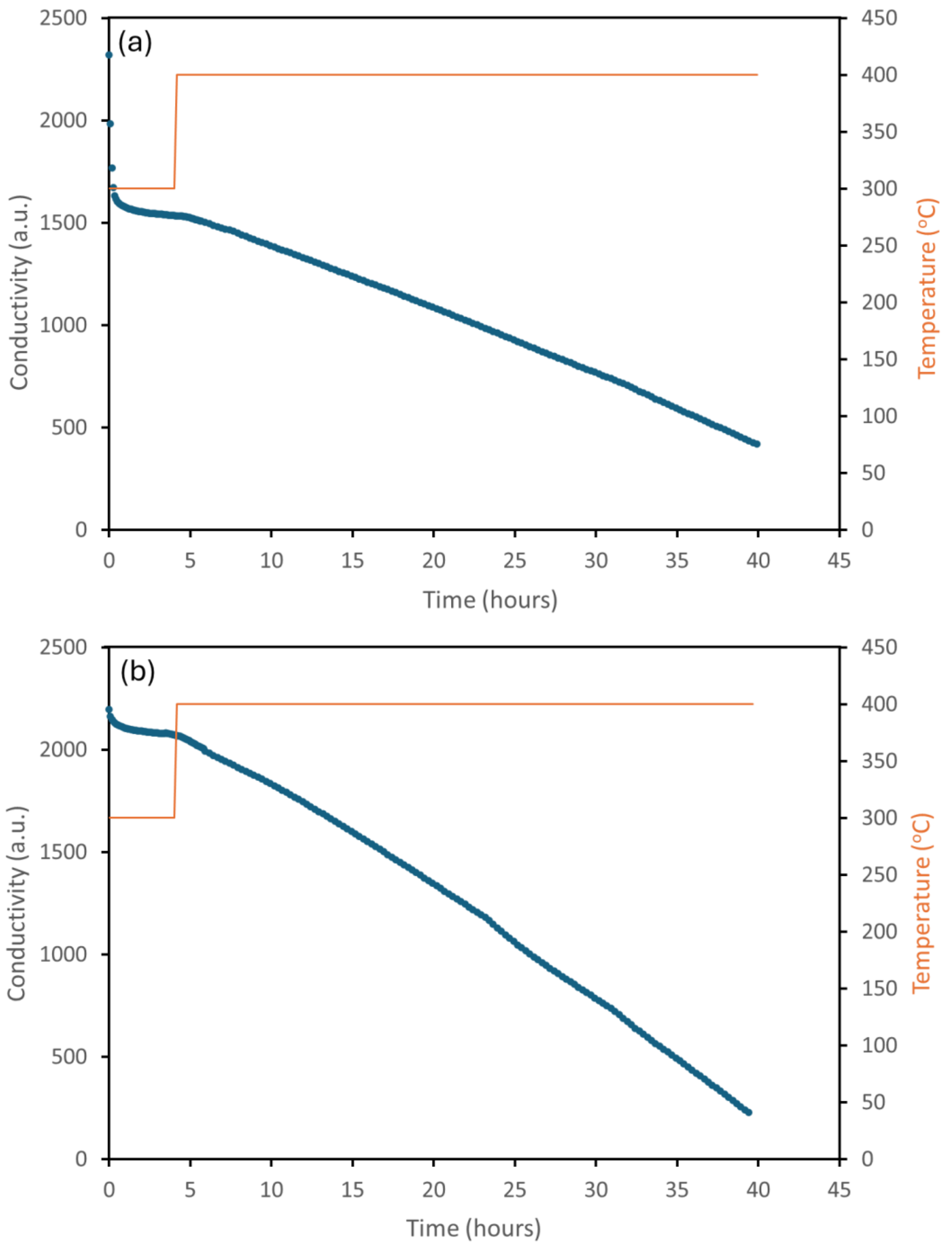

Figure S8: Conductivity profiles for the materials reacted with 3:1 H_2/N_2 at 300 °C for 4 h and 400 °C for 35 h 30 min, related to Table 3 and Figure 5. A.) $Ru_{1.03}Fe_3N_{1.08}$-500 °C -24 h. B.) $Ru_{0.82}Fe_3N_{0.78}$-600 °C -12 h.

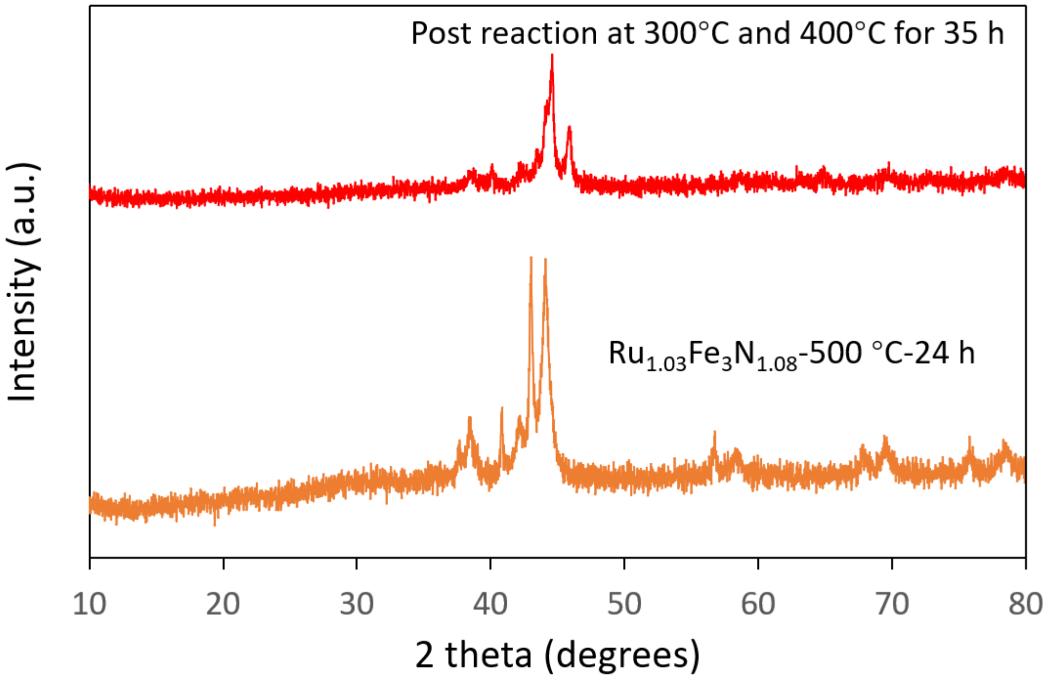

Figure S9: XRD patterns of $Ru_{1.03}Fe_3N_{1.08}$-500 °C-24 h before and after ammonia synthesis reaction, related to Figure 6. A.) pre-reaction. B.) post-reaction at 300 °C for 4 h and 400 °C for 35 h 30 min.

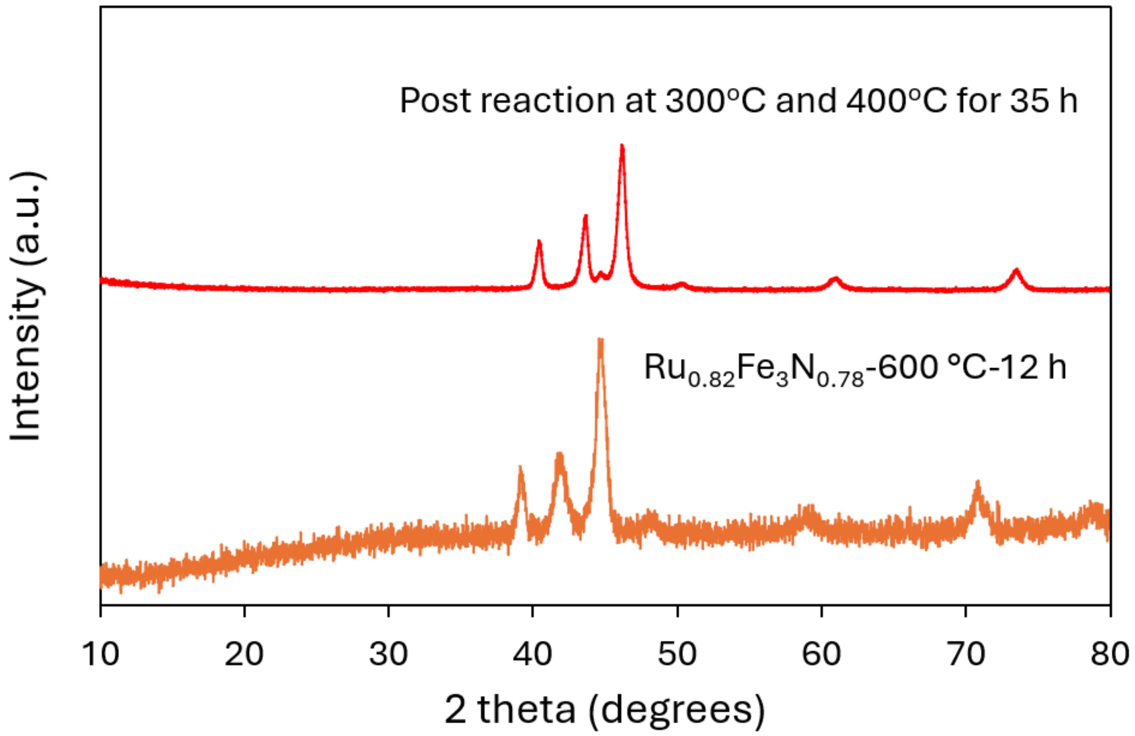

Figure S10: XRD patterns of $Ru_{0.82}Fe_3N_{0.78}$-600 °C-12 h before and after ammonia synthesis reaction, related to Figure 6. A.) pre-reaction. B.) post-reaction at 300 °C for 4 h and 400 °C for 35 h 30 min.

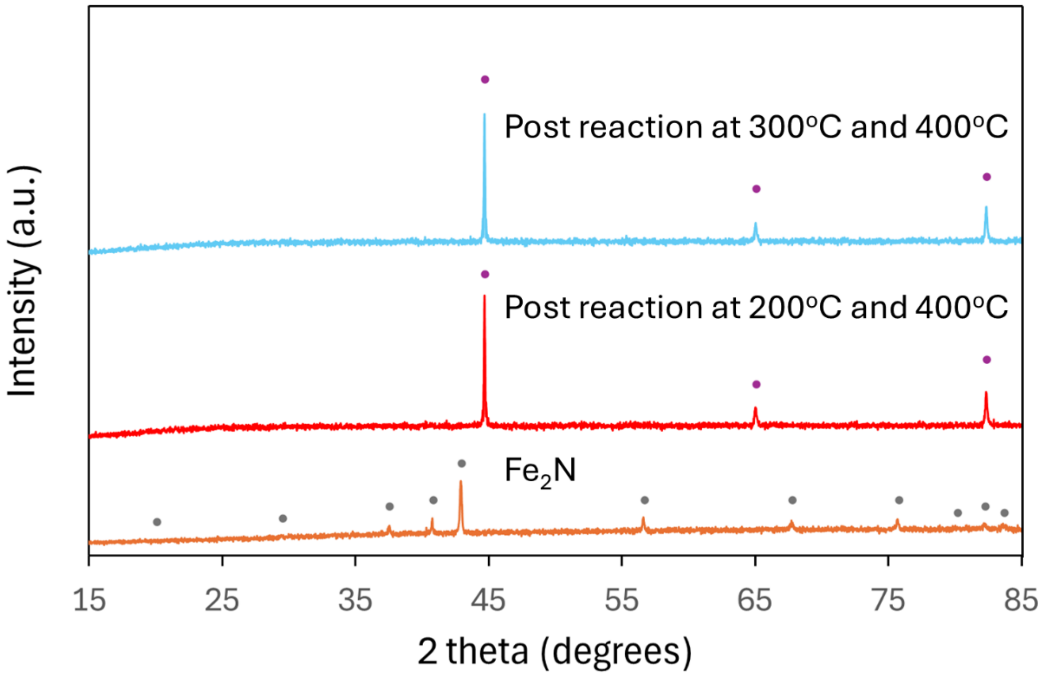

Figure S11: XRD patterns of Fe_2N before and after ammonia synthesis reaction, related to Figure 7. A.) pre-reaction. B.) post-reaction at 200 °C and 400 °C. C.) post-reaction at 300 °C and 400 °C. ● Fe_2N and ● Fe.

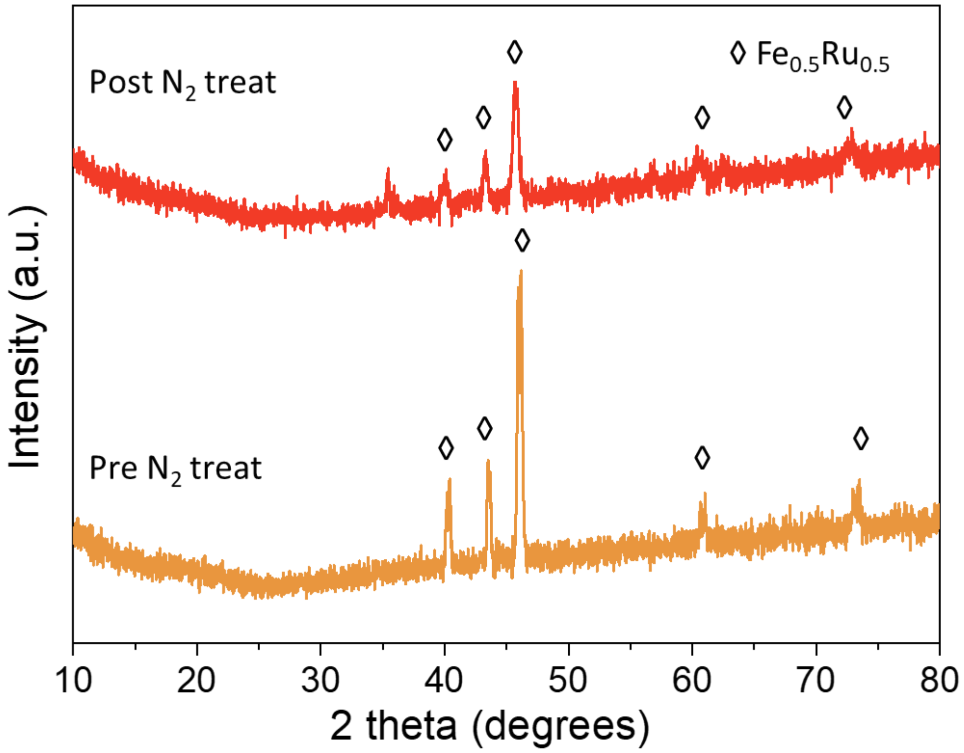

Figure S12: XRD patterns for $Ru_{0.86}Fe_3N_{0.80}$-900 °C-12h pre- and post-treatment with N_2, related to Figure 9. A.) pre-treatment. B.) post-treatment at 700 °C for 4 hours.

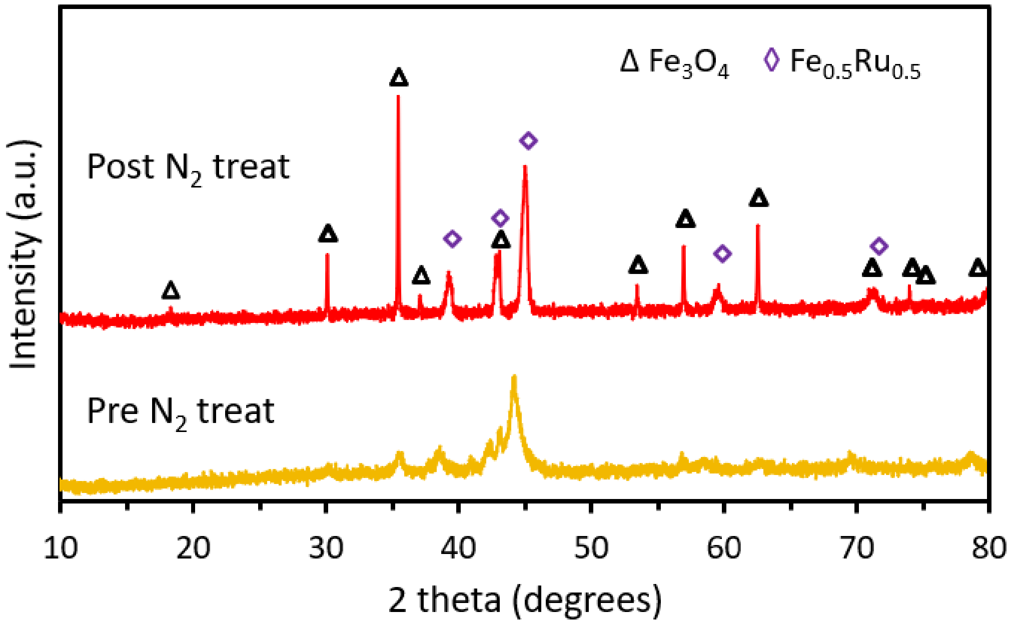

Figure S13: XRD patterns for $Ru_{1.03}Fe_3N$-500 °C-24 h pre- and post-treatment with N_2, related to Figure 9. A.) pre-treatment. B.) post-treatment at 700 °C for 4 hours.

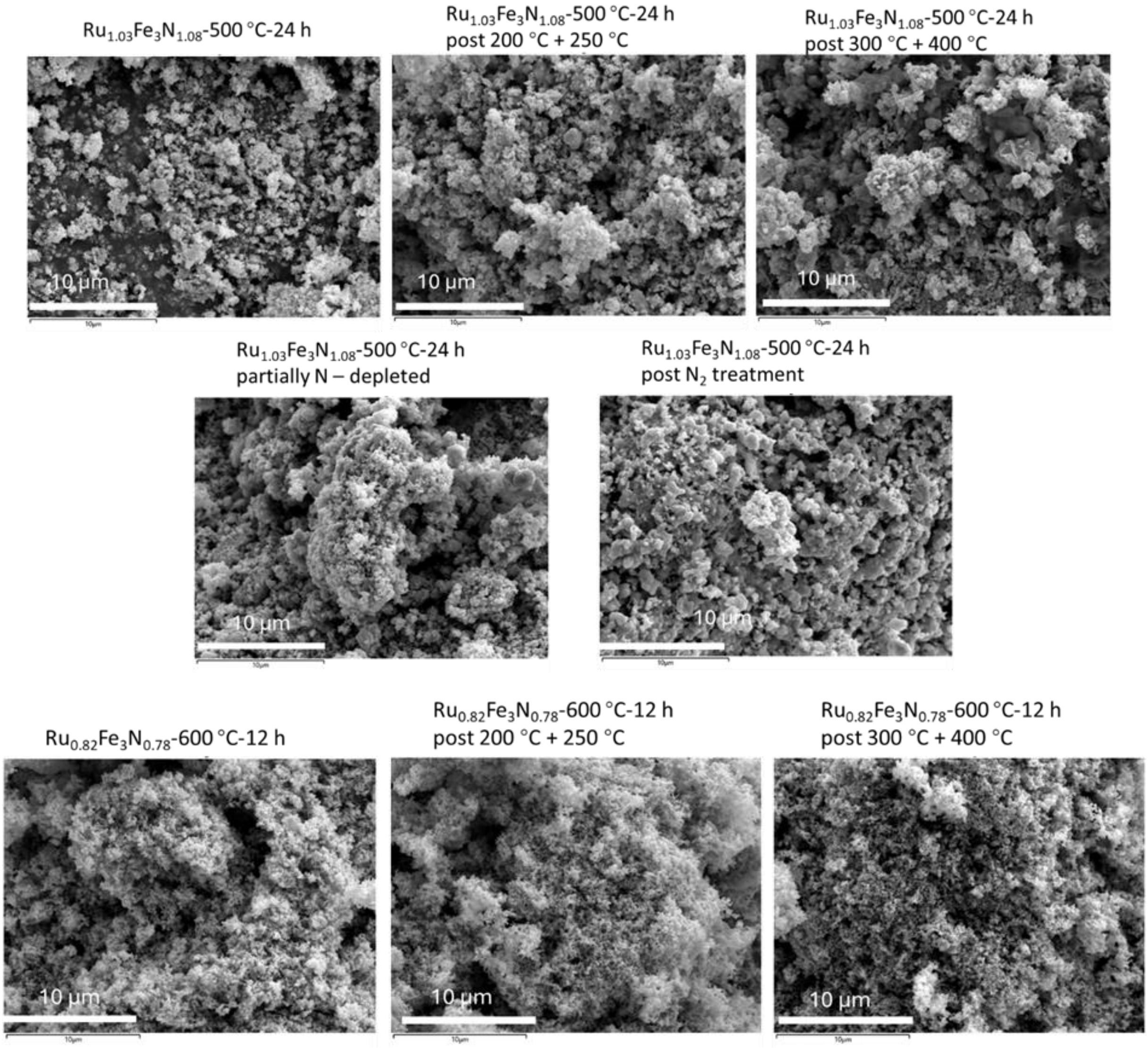

Figure S14: SEM images of sample Ru$_{1.03}$Fe$_3$N$_{1.08}$-500 °C-24 h after reaction at 200 -400 °C and after regeneration by N_2 treatment, and sample Ru$_{0.8}$Fe$_3$N$_{0.78}$-600 °C-12 h after reaction at 200 -400 °C, related to Figure 1.

Table S1: Synthetic process of ruthenium iron nitrides, nitrogen mass fractions in samples and sample compositions expressed as Ru$_x$Fe$_3$N$_y$, related to Table 1.

**Sample No.**	**Ru: Fe molar ratio in solution**	**Ammonolysis**		**Nitrogen content (wt. %)**	**Composition expressed as Ru$_x$Fe$_3$N$_y$**
		**Temperature/°C**	**Duration/h**		
1	1.4:3	500 °C	12 h	4.36%	Ru$_{1.03}$Fe$_3$N$_{0.88}$
2	1.4:3	500 °C	168 h	3.91%	Ru$_{1.15}$Fe$_3$N$_{0.82}$

*Samples are referred to Ru$_x$Fe$_3$N$_y$, followed by the ammonolysis temperature and duration.

Table S2: Lattice parameters, phase ratio and nitrogen contents of the $Ru_xFe_3N_y$ samples prepared under different ammonolysis temperatures, related to Table 1 and Figure 2.

**Sample Name**	**Ammonolysis temperature ℃**	**Lattice parameters (Å); phase ratio**	**Nitrogen content (wt. %)**
$Ru_{1.12}Fe_3N_{1.23}$-400 °C-24 h	400 °C	a=4.66; c=4.29; 0.50	5.78%
		a=4.79; c=4.41; 0.50	
$Ru_{1.03}Fe_3N_{1.08}$-500 °C-24 h	500 °C	a=4.68; c=4.30; 0.57	5.26%
		a=4.78; c=4.43; 0.43	
$Ru_{1.07}Fe_3N_{0.53}$-600 °C-24 h	600 °C	a=4.59; c=4.27	2.60%
$Ru_{1.13}Fe_3N_{0.49}$-700 °C-24 h	700 °C	a=4.60; c=4.28	2.40%
$Ru_{1.04}Fe_3N_{0.50}$-800 °C-24 h	800 °C	a=4.60; c=4.28	2.49%
$Ru_{0.96}Fe_3N_{0.52}$-900 °C-24 h	900 °C	a=4.61; c=4.29	2.69%

Table S3: Ru and Fe molar ratio in solution, lattice parameters, phase ratio and nitrogen contents of the $Ru_xFe_3N_y$ samples prepared from the solution with different ruthenium and iron molar ratios and ammonolysis temperatures, related to Table 1.

**Sample Name**	**Ru: Fe molar ratio in solution**	**Lattice parameters(Å); phase ratio**	**Nitrogen content (wt. %)**
$Ru_{0.77}Fe_3N_{0.97}$-500 °C-24 h	1.0:3	a=4.64; c=4.35;	5.13%
$Ru_{1.03}Fe_3N_{1.07}$-500 °C-24 h	1.4:3	a=4.62; c=4.28; 0.72	5.26%
		a=4.75; c=4.40; 0.28	
$Ru_{1.20}Fe_3N_{0.79}$-500 °C-24 h	1.5:3	a=4.62; c=4.27; 0.74	3.70%
		a=4.68; c=4.30; 0.26	
$Ru_{1.96}Fe_3N_{0.82}$-500 °C-24 h	1.6:3	a=4.61; c=4.26; 0.74	3.05%
		a=4.66; c=4.28; 0.26	
$Ru_{0.51}Fe_3N_{1.07}$-900 °C-12 h	0.6:3	a=4.70; c=4.38	6.42%
$Ru_{0.86}Fe_3N_{0.80}$-900 °C-12 h	1.0:3	a=4.67; c=4.36	4.22%
$Ru_{0.93}Fe_3N_{0.56}$-900 °C-12 h	1.4:3	a=4.60; c=4.28	2.90%
$Ru_{1.11}Fe_3N_{0.42}$-900 °C-12 h	1.6:3	a=4.59; c=4.26	2.07%

Table S4: Lattice parameters, phase ratio and nitrogen contents of the $Ru_xFe_3N_y$ samples prepared under different ammonolysis durations, related to Table 1.

**Sample Name**	**Ammonolysis time (hours)**	**Lattice parameters (Å); phase ratio**	**Nitrogen content (wt. %)**
$Ru_{1.03}Fe_3N_{0.88}$-500 °C-12 h	12	a=4.65; c=4.29; 0.82	4.36 %
		a=4.76; c=4.41; 0.18	
$Ru_{1.03}Fe_3N_{1.07}$-500 °C-24 h	24	a=4.68; c=4.30; 0.57	5.26 %
		a=4.78; c=4.43; 0.43	
$Ru_{1.15}Fe_3N_{0.82}$-500 °C-168 h	168	a=4.63; c=4.29; 0.90	3.91 %
		a=4.77; c=4.42; 0.10	

Table S5: The results of Rietveld refinements of $Ru_{1.03}Fe_3N_{1.08}$-500°C-24h fitted with two ε-Fe_3N type $P6_322$., related to Table 2. A.) The space groups, lattice parameters and phase fractions of $Ru_{1.03}Fe_3N_{1.08}$-500°C-24h. B.) The atom positions, fractions and Uiso in Phase 1 $P6_322$. C.) The atom positions, fractions and Uiso in Phase 2 $P6_322$.

(a)

	Space group	Lattice parameters			Phase fraction
		a	c	Volume	
Phase 1	$P6_322$	4.67	4.30	81.28	44.0%
Phase 2	$P6_322$	4.77	4.42	87.26	56.0%

(b) The atom positions, fractions and Uiso in Phase 1 $P6_322$:

**Name**	**x**	**y**	**z**	**Fraction**	**Uiso**
Fe	0.32943	0.00000	0.00000	0.7000	0.00333
N(1)	0.33330	0.66670	0.25000	0.0000	0.01515
Ru	0.32943	0.00000	0.00000	0.3000	0.00333
N(2)	0.00000	0.00000	0.25000	0.4269	0.01515

(c) The atom positions, fractions and Uiso in Phase 2 $P6_322$:

**Name**	**x**	**y**	**z**	**Fraction**	**Uiso**
Fe	0.34054	0.00000	0.00000	0.7000	0.00441
N(1)	0.33330	0.66670	0.25000	0.7664	0.00193
Ru	0.34054	0.00000	0.00000	0.3000	0.00441
N(2)	0.00000	0.00000	0.25000	0.4529	0.00193

Table S6: The results of Rietveld refinements of $Ru_{1.03}Fe_3N_{1.08}$-500 °C-24 h fitted with ε-Fe_3N type $P6_322$ structure and $P6_3/mmc$ structure, related to Figure 3. The space groups, lattice parameters and phase fractions are shown in Table 2. A.) The atom positions, fractions and Uiso in Phase 1 $P6_322$. B.) The atom positions, fractions and Uiso in Phase 2 $P6_3/mmc$.

(a) The atom positions, fractions and Uiso in Phase 1 $P6_322$:

**Name**	**x**	**y**	**z**	**Fraction**	**Uiso**
Fe	0.34578	0.00000	0.00000	0.7000	0.00395
N(1)	0.33330	0.66670	0.25000	0.7778	0.00341
Ru	0.34578	0.00000	0.00000	0.3000	0.00395
N(2)	0.00000	0.00000	0.25000	0.4667	0.00341
N(3)	0.33330	0.66670	0.75000	0.0355	0.00341

(b) The atom positions, fractions and Uiso in Phase 2 $P6_322$:

**Name**	**x**	**y**	**z**	**Fraction**	**Uiso**
Fe	0.33330	-0.33330	0.25000	0.7000	0.00369
N(1)	0.00000	0.00000	0.00000	0.1233	0.00312
Ru	0.33330	-0.33330	0.25000	0.3000	0.00369

Table S7: Nitrogen analysis of $Ru_{1.03}Fe_3N$-500 °C-24 h pre- and post-reaction with 3:1 H_2/N_2 at 200 °C for 30 minutes and post N_2 treatment at 700 °C for 4 hours, related to Figure S13.

Material	Nitrogen content (wt. %)
Ru-Fe-N (1.4:3)	5.22
Ru-Fe-N post-reaction	1.12
Ru-Fe-N post N_2 treatment	0.00

Table S8: EDX compositional analysis of samples $Ru_{1.03}Fe_3N_{1.08}$-500 °C-24 h and $Ru_{0.8}Fe_3N_{0.78}$-600 °C-12 h before and after ammonia synthesis reaction and after N_2 treatment, related to Figure 1.

**Sample Name**	**Ru (wt. %)**	**Fe (wt. %)**	**N (wt. %)**	**O (wt. %)**	**Ru: Fe (wt.%)**
$Ru_{1.03}Fe_3N_{1.08}$-500 °C-24 h	35	59	5	-	1.77:3
$Ru_{1.03}Fe_3N_{1.08}$-500 °C-24 h post 200 °C + 250 °C	43	57	-	-	2.26:3
$Ru_{1.03}Fe_3N_{1.08}$-500 °C-24 h post 300 °C + 400 °C	38	62	-	-	1.84:3
$Ru_{1.03}Fe_3N_{1.08}$-500 °C-24 h partially N – depleted	39	57	4	-	1.89:3
$Ru_{1.03}Fe_3N_{1.08}$-500 °C-24 h post N_2 treatment	35	52	-	13	2.01:3
$Ru_{0.82}Fe_3N_{0.78}$-600 °C-12 h	30	64	7	-	1.41:3
$Ru_{0.82}Fe_3N_{0.78}$-600 °C-12 h post 200 °C + 250 °C	33	67	-	-	1.48:3
$Ru_{0.82}Fe_3N_{0.78}$-600 °C-12 h post 300 °C + 400 °C	33	67	-	-	1.48:3
